# Anisotropic exchange interaction of two hole-spin qubits

**DOI:** 10.1038/s41567-024-02481-5

**Published:** 2024-05-06

**Authors:** Simon Geyer, Bence Hetényi, Stefano Bosco, Leon C. Camenzind, Rafael S. Eggli, Andreas Fuhrer, Daniel Loss, Richard J. Warburton, Dominik M. Zumbühl, Andreas V. Kuhlmann

**Affiliations:** 1 Department of Physics, University of Basel, Basel, Switzerland; 2 IBM Research Europe-Zurich, Rüschlikon, Switzerland; 3 Present Address: RIKEN, Center for Emergent Matter Science (CEMS), Wako-shi, Japan

**Keywords:** Quantum dots, Qubits, Quantum information

## Abstract

Semiconductor spin qubits offer the potential to employ industrial transistor technology to produce large-scale quantum computers. Silicon hole spin qubits benefit from fast all-electrical qubit control and sweet spots to counteract charge and nuclear spin noise. However, the demonstration of a two-qubit interaction has remained an open challenge. One missing factor is an understanding of the exchange coupling in the presence of a strong spin–orbit interaction. Here we study two hole-spin qubits in a silicon fin field-effect transistor, the workhorse device of today’s semiconductor industry. We demonstrate electrical tunability of the exchange splitting from above 500 MHz to close-to-off and perform a conditional spin-flip in 24 ns. The exchange is anisotropic because of the spin–orbit interaction. Upon tunnelling from one quantum dot to the other, the spin is rotated by almost 180 degrees. The exchange Hamiltonian no longer has the Heisenberg form and can be engineered such that it enables two-qubit controlled rotation gates without a trade-off between speed and fidelity. This ideal behaviour applies over a wide range of magnetic field orientations, rendering the concept robust with respect to variations from qubit to qubit, indicating that it is a suitable approach for realizing a large-scale quantum computer.

## Abstract (web summary)

A successful silicon spin qubit design should be rapidly scalable by benefiting from industrial transistor technology. This investigation of exchange interactions between two FinFET qubits provides a guide to implementing two-qubit gates for hole spins.

## Main

Semiconductor quantum dot (QD) spin qubits are prime candidates for future implementations of large-scale quantum circuits^1–3^. Currently, the most advanced spin-based quantum processor allows for universal control of six electron spin qubits in silicon (Si)^4^, closely followed by a four-qubit demonstration with holes in germanium^5^. In comparison to electron spins, hole spins have the advantage that they can be controlled all-electrically, without the added complexity of on-chip micromagnets^6,7^ or the need for orbital degeneracy^8^, thanks to their intrinsic spin–orbit interaction (SOI). Moreover, holes benefit from a reduced hyperfine interaction^9^ and the absence of valleys^10^.

Holes in quasi-one-dimensional (1D) nanostructures are highly attractive for implementing fast and coherent qubits. The mixing of heavy- and light-hole states on account of the 1D-confinement results in an unusually strong and electrically tunable direct Rashba SOI, with sweet spots for charge and hyperfine noise^11–13^, enabling ultra-fast hole spin qubits^14,15^ with reduced sensitivity to noise^16^. Conveniently, such a 1D-system can be realized using today’s industry standard transistor design known as the fin field-effect transistor (FinFET)^17^. Adapting FinFETs for QD integration^16,18–22^ potentially facilitates quantum computer scale-up by leveraging decades of technology development in the semiconductor industry^23^. Furthermore, recent research has shown that individual hole spin qubits in a bulk-Si FinFET can be operated at temperatures above 4 K (ref. ^22^), paving the way for FinFET-based quantum integrated circuits that host both the qubit array and its classical control electronics on the same chip^24–26^.

Universal quantum computation requires both single-qubit control and two-qubit interactions. Native two-qubit gates for spins such as the $\sqrt{{{{\rm{SWAP}}}}}$ (refs. ^1,27^), the controlled phase^28–31^ or the controlled rotation (CROT)^4,5,24,29,32–35^ rely on the exchange interaction that arises from the wavefunction overlap between two adjacent QDs. For electrons in Si, two-qubit gate fidelities have recently surpassed 99% (refs. ^30,31,34^), but for holes in Si or FinFETs, the demonstration of two-qubit logic is still missing due to the challenges in obtaining a controllable exchange interaction^36^.

We make an important step towards a FinFET-based quantum processor by demonstrating control over the exchange of two holes in a Si FinFET. While the exchange interaction is crucial for implementing high-fidelity two-qubit gates, it is, particularly for hole spins, still largely unexplored. We measure the dependence of the exchange splitting on the magnetic field direction and find large values in some directions but close-to-zero values in other directions. In addition, we develop a general theoretical framework applicable to a wide range of devices and identify the SOI as the main reason for the exchange anisotropy. From our measurements, we can extract the full exchange matrix and hence accurately determine the Hamiltonian of the two coupled spins, allowing us to predict the optimum operating points for the gates. For holes, unlike electrons, the strong exchange anisotropy facilitates CROTs with both high fidelity and high speed, for an experimental setting that is robust against device variations.

**Fig. 1:**
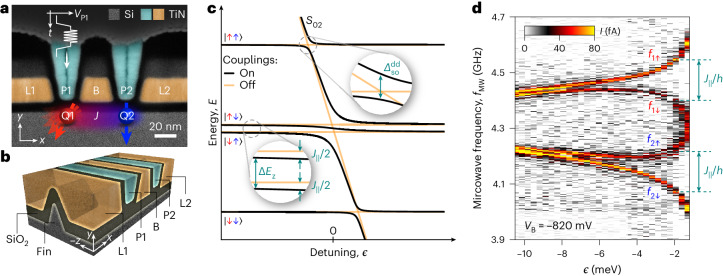
Two-qubit system in a Si FinFET. **a**, False-colour transmission electron microscope image of a co-fabricated device showing the cross-section along the fin. The qubits (Q1, Q2) are located underneath the plunger gates (P1, P2) and are manipulated by applying microwaves to the P1-gate. The barrier gate (B) controls the interdot tunnelling; the lead gates (L1, L2) accumulate the hole reservoirs. Measurements are performed on a device with ≃20 nm-wide B- and P-gates. **b**, A three-dimensional render of the device, illustrating the triangular-shaped fin covered by the wrap-around gates. **c**, Two-spin energy-level diagram close to the (1,1)–(0,2) charge transition with (black) and without (orange) interactions. A *g*-tensor mismatch yields the singlet–triplet mixed states $\left\vert \uparrow \downarrow \right\rangle ,\left\vert \downarrow \uparrow \right\rangle$. The singlet state *S*_02_ hybridizes with the antiparallel (parallel) two-spin states on account of spin-conserving tunnelling (SOI). A finite exchange splitting *J*_∥_ lowers the energy of the antiparallel two-spin states with respect to the parallel ones. **d**, Exchange spin funnel measurements for both qubits, revealing an increase (decrease) in *f*_1*↑*_, *f*_2*↑*_ (*f*_1*↓*_, *f*_2*↓*_) at the upper (lower) branch. Data was taken at *V*_B_ = −820 mV and ∣**B**∣ = 0.146 T with orientation *α* = 30°, *β* = 0°. Source data

Figure 1a shows the device cross-section along the triangular-shaped fin, revealing ultrashort lengths, highly uniform profiles and perfect alignment of the gate electrodes^19,20^; Fig. 1b presents a three-dimensional illustration of the device. The double quantum dot (DQD) hosting qubits Q1 and Q2 is formed beneath plunger gates P1 and P2, and the barrier gate B provides control over the interdot tunnel coupling *t*_c_ (ref. ^22^). The distance between the QDs was chosen to match the spin–orbit length^20,22^. Taking advantage of the strong SOI, all-electrical spin control is implemented by electric-dipole spin resonance (EDSR)^37,38^. For this purpose, fast voltage pulses and microwave (MW) bursts are applied to P1 and a spin-flip is detected in the form of an increased spin blockade leakage current. The device is tuned close to the (1,1)–(0,2) charge transition, where (*n*, *m*) denotes a state with *n* (*m*) excess holes on the left (right) QD. In Fig. 1c, the eigenenergies of the two-spin states ($\,\left\vert \uparrow \uparrow \right\rangle ,\,\left\vert \uparrow \downarrow \right\rangle ,\,\left\vert \downarrow \uparrow \right\rangle ,\,\left\vert \downarrow \downarrow \right\rangle \,$) in the (1,1) and the singlet ground state *S*_02_ in the (0,2) charge region are plotted as a function of the detuning *ϵ*, which describes the energy difference between the (1,1) and (0,2) charge states. While spin-conserving tunnelling causes an anticrossing between the *S*_02_ and the antiparallel two-spin states, non-spin-conserving tunnelling due to the SOI results in an anticrossing between the *S*_02_ and the parallel two-spin states. As a consequence of the anticrossing with the singlet state, the energy of the antiparallel states decreases by *J*_∥_(*ϵ*)/2, where *J*_∥_(*ϵ*) is the measured exchange splitting between the two spins. The energy-level structure of the two hole system can be probed by performing MW spectroscopy (Fig. 1d): at large negative *ϵ*, the resonance frequencies of both qubits differ due to the individual *g*-tensor ${\hat{g}}_{i}$ for each QD and are independent of each other. At more positive detunings, closer to the (0,2) region, the exchange interaction splits both resonances by *J*_∥_/*h* (*h* denotes Planck’s constant), resulting in four conditional transitions. The corresponding EDSR frequencies are denoted by *f*_*iσ*_, where *i* is the index of the target qubit and *σ* the control qubit state, $\left\vert \uparrow \right\rangle$ or $\left\vert \downarrow \right\rangle$.

**Fig. 2:**
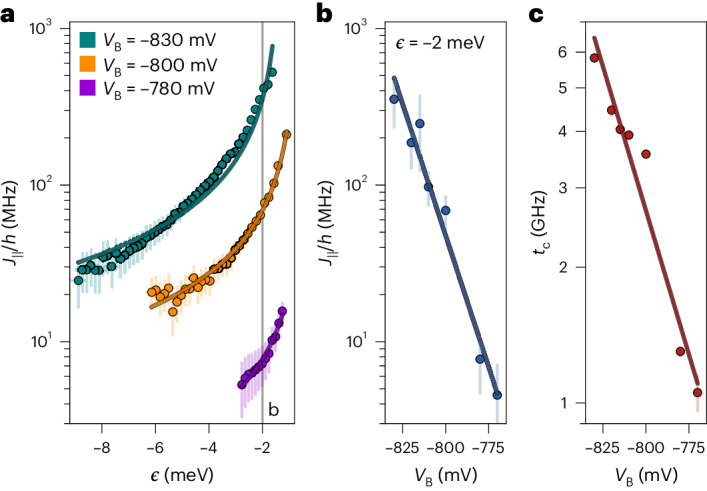
Tunable exchange coupling. **a**, Detuning dependence of the exchange frequency for *V*_B_ = −830, −800 and −780 mV. The solid curves represent fits to equation (1) and errors represent the width of the EDSR resonance. **b**,**c**, *J*_∥_/*h* for *ϵ* = −2 meV (**b**) and tunnel coupling (**c**) as functions of *V*_B_, both determined from fits, as shown in **a**. The solid lines show exponential function fits to the data. The error bars in **b** represent the estimated errors due to a detuning uncertainty, and in **c** represent the standard errors for the best-fit values. Fitted *U*_0_ values are provided in Supplementary Section 8. Source data

We map out the *ϵ*-dependence of *J*_∥_ that, as shown in Fig. 2a, is well described by

1 $${J}_{\parallel }={J}_{0}\cos (2\tilde{\theta })=\frac{2{t}_{\rm{c}}^{2}}{{U}_{0}-\epsilon }\cos (2\tilde{\theta }),$$

valid in the limit of *t*_c_ ≪ *U*_0_ − *ϵ* (refs. ^39–41^). Here, *U*_0_ is an energy offset of the *ϵ* axis, *J*_0_ the bare exchange and $\cos (2\tilde{\theta })$ an SOI-induced correction factor, which is discussed later. The exchange splitting shows an exponential dependence on the barrier gate voltage *V*_B_ (Fig. 2b) and reaches values of up to ~525 MHz. At the same time, exchange can be turned off within the resolution limit of our spectroscopy experiment that is given by the EDSR linewidth of ≃2 MHz (refs. ^29,33,41^). This means, using the two ‘control knobs’ *ϵ* and *V*_B_, we achieve excellent control over the exchange coupling. As ${t}_{\rm{c}}\propto {J}_{\parallel }^{1/2}$, the tunnel coupling is also exponentially dependent on *V*_B_ and tunable by almost one order of magnitude (Fig. 2c).

**Fig. 3:**
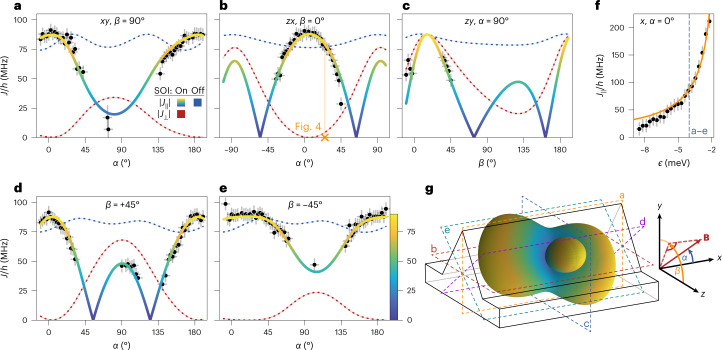
Anisotropic exchange. **a**–**e**, Exchange frequency as a function of magnetic field direction, which is expressed with the angles *α* and *β* (see coordinate system in **g**), for five different planes at *ϵ* = −4.03 meV: *α* varies, *β* = 90° (**a**); *α* varies, *β* = 0° (**b**); *α* = 90°, *β* varies (**c**); *α* varies, *β* = 45° (**d**); and *α* varies, *β* = −45° (**e**). For certain **B** orientations, the qubits could not be read out via Pauli spin blockade and hence *J*_∥_/*h* (black points) could not be determined. **f**, Detuning dependence of *J*_∥_/*h* for **B** applied in the *x* direction. The multicoloured curves in **a**–**e** and the orange one in **f** represent a common fit of equation (3) to all the data presented in this figure. While the red dashed curves in **a**–**e** visualize ∣*J*_⊥_∣/*h*, the blue dashed ones illustrate the exchange modulation due to the different and anisotropic *g*-tensors in the absence of SOI. **g**, Schematic representation of the fin structure (black and grey lines) overlaid by a 3D surface plot of ∣*J*_∥_∣/*h*. The coloured dashed rectangles indicate the planes of **a**–**e**. The data presented in this figure are taken at *V*_B_ = −820 mV and the error bars account for the EDSR linewidth and uncertainties in **B** field due to magnetic flux trapping. Source data

In Fig. 3a–e, the dependence of *J*_∥_ on the magnetic field orientation is shown, revealing a striking anisotropy with vanishing splittings. The highly anisotropic exchange frequency is mainly due to the strong SOI and can be qualitatively understood from the gap size ${\Delta }_{{{{\rm{so}}}}}^{{{{\rm{dd}}}}}$ of the anticrossing between the *S*_02_ and the parallel two-spin states. ${\Delta }_{{{{\rm{so}}}}}^{{{{\rm{dd}}}}}$ is proportional to $\left| \hat{\mathbf{n}}_{\mathrm{so}}\times \,\mathbf{B} \right|$, where **B** is the external magnetic field and ${\hat{\bf{n}}_{\rm{so}}}$ a unit vector pointing in the direction of the spin–orbit field^42^. We expect ${\hat{\bf{n}}_{\rm{so}}}\,\propto \,\mathbf{k}\times \mathbf{E}$ with momentum operator **k** and applied electric field **E** (ref. ^11^). Therefore, ${\Delta }_{{{{\rm{so}}}}}^{{{{\rm{dd}}}}}$ changes with magnetic field orientation and so do the two hole energy levels (see Fig. 1c). However, we remark that from the dependence of ${\Delta }_{{{{\rm{so}}}}}^{{{{\rm{dd}}}}}$ on **B**/∣**B**∣, which is extracted close to zero detuning, the exchange matrix $\hat{{{{\mathcal{J}}}}}$ at the qubit operation point cannot be extracted due to the voltage dependence of both the *g*-tensors and the SOI.

We derive an equation for $\hat{{{{\mathcal{J}}}}}$ starting from a Fermi–Hubbard model and including both the SOI and the anisotropic and differing hole *g*-tensors (Methods and Supplementary Section 5). Tuned deeply into the (1,1) charge regime where spin manipulation takes place, the system is approximated by the Hamiltonian

2 $${H}_{(1,1)}=\frac{1}{2}{\mu }_{\rm{B}}{\bf{B}}\cdot {\hat{g}}_{1}\cdot {\mathbf{\upsigma }}_{1}+\frac{1}{2}{\mu }_{\rm{B}}{\bf{B}}\cdot {\hat{g}}_{2}\cdot {\mathbf{\upsigma }}_{2}+\frac{1}{4}{\boldsymbol{\sigma }}_{1}\cdot {\hat{\mathcal{J}}}\cdot {\mathbf{\upsigma }}_{2}.$$

Here, *μ*_B_ is Bohr’s magneton and **σ**_*i*_ the vector of Pauli matrices for each QD. The exchange matrix is given by $\hat{{{{\mathcal{J}}}}}={J}_{0}{\hat{R}}_{{{{\rm{so}}}}}(-2d/{\lambda }_{{{{\rm{so}}}}})$, where ${\hat{R}}_{{{{\rm{so}}}}}(\varphi )$ is the counterclockwise rotation matrix around ${\hat{\bf{n}}}_{\rm{so}}$ by an angle *φ*, *λ*_so_ is the spin–orbit length and *d* is the interdot distance. We use the convention that displacing a spin by π*λ*_so_/2 induces a spin rotation of π (ref. ^43^). The experimentally observed exchange splitting is given by (Methods and Supplementary Section 5):

3 $${J}_{\parallel }={{{{\hat{\bf{n}}}}}}_{1}\cdot \hat{{{{\mathcal{J}}}}}\cdot {{{{\hat{\bf{n}}}}}}_{2}={J}_{0}{{{{\hat{\bf{n}}}}}}_{1}\cdot {\hat{R}}_{{{{\rm{so}}}}}(-2d/{\lambda }_{{{{\rm{so}}}}})\cdot {{{{\hat{\bf{n}}}}}}_{2},$$

where ${{{{\hat{\bf{n}}}}}}_{i}={\hat{g}}_{i}\cdot {{{\bf{B}}}}/| {\hat{g}}_{i}\cdot {{{\bf{B}}}}|$ denotes the Zeeman field direction. On comparing equations (1) and (3), we find for the previously introduced correction factor $\cos (2\tilde{\theta })={{{{\hat{\bf{n}}}}}}_{1}\cdot {\hat{R}}_{{{{\rm{so}}}}}(-2d/{\lambda }_{{{{\rm{so}}}}})\cdot {{{{\hat{\bf{n}}}}}}_{2}$. Finally, by describing the magnetic field direction using the two angles *α* and *β* (Fig. 3), we obtain a fit equation *J*_∥_(*α*, *β*) with five fitting parameters, namely *t*_c_, *U*_0_, ${\hat{\bf{n}}}_{\rm{so}}$ and *λ*_so_.

Next, we apply this model to the data (black points) shown in Fig. 3a–f and perform a common fit to the full data set, consisting of measurements of *J*_∥_(*α*, *β*) in five different planes (visualized in Fig. 3g) at constant detuning and for *J*_∥_(*ϵ*) for **B** pointing in the *x* direction. There is excellent agreement between theory and experiment for the best-fit parameters: *λ*_so_ = 31 nm, $\hat{\bf{n}}_{\rm{so}}$ = (−0.06, 0.41, 0.91), *t*_c_ = 5.61 GHz and *U*_0_ = 1.07 meV. The spin–orbit length coincides with the values reported previously^20,22^ and corresponds to a spin rotation angle of 2*θ*_so_ = 2*d*/*λ*_so_ ≈ 0.82π for a hole tunnelling from one QD to the other over *d* ≈ 40 nm. The direction of the spin–orbit field, represented by (*α*_so_ = 93°, *β*_so_ = 23°) is, as expected, perpendicular to the long axis of the fin and thus orthogonal to the hole momentum^11,13^. The small out-of-the-substrate-plane tilt can arise on account of strain or electric fields not being perfectly aligned along the *y* direction. Using the five best-fit parameter values, we can reconstruct the full exchange matrix

4 $$\hat{{{{\mathcal{J}}}}}={J}_{0}\left(\begin{array}{ccc}-0.87&0.41&-0.28\\ -0.49&-0.60&0.64\\ 0.10&0.69&0.72\end{array}\right).$$

Because we also find the *g*-tensors when measuring *J*_∥_(*α*, *β*) by means of MW spectroscopy, the two-spin Hamiltonian (equation (2)) is fully characterized, thus allowing us to optimize two-qubit gate operations, as discussed later. Furthermore, we can analyse the different contributions to the exchange anisotropy with equation (3): by setting *θ*_so_ to zero, we are left with the effect of the anisotropic *g*-tensors. We find that the *g*-tensor contribution to the *J*_∥_-anisotropy was minor (dashed blue curves in Fig. 3a–e). Finally, we remark that the observed rotational exchange anisotropy relies on a strong SOI and the presence of an external magnetic field^44,45^, as opposed to a weaker Ising-like anisotropy that can be found in inversion symmetric hole DQDs^46^ or at zero magnetic field^47,48^.

**Fig. 4:**
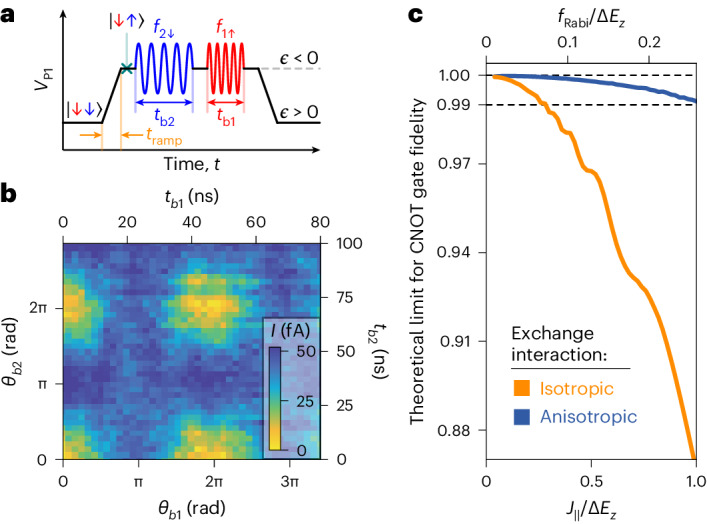
Fast two-qubit CROTs for Si hole spin qubits. **a**, Pulse sequence for the CROT operation. A trapezoidal waveform with a ramp time of 20 ns is used to initialize the spins in the $\left\vert \downarrow \uparrow \right\rangle$-state and to read out their state after applying two microwave bursts resonant with Q2 (*f*_2↓_ = 4.25 GHz) and Q1 (*f*_1↑_ = 4.66 GHz). **b**, Parity measurement of the qubits demonstrating a conditional rotation of Q1 controlled by the state of Q2. This data is taken at *J*_∥_/*h* ≈ 80 MHz, *V*_B_ = −810 mV, ∣**B**∣ = 0.146 T, *α* = 25°, *β* = 0° and *ϵ* = −2.9 meV. **c**, Numerically calculated CNOT gate fidelity versus exchange splitting *J*_∥_ (bottom *x* axis) and ${f}_{{{{\rm{Rabi}}}}}={J}_{\parallel }/\sqrt{15}/h$ (top *x* axis, both axes in units of Δ*E*_Z_) for anisotropic exchange, with parameters as in **b** (blue) and isotropic exchange (orange). The shaded regions indicate the precision of the numerics. Source data

We make use of the large exchange splitting to demonstrate a fast two-qubit CROT^4,5,24,29,32–35^ for holes in Si. This quantum operation is naturally implemented by driving just one of the four EDSR transitions (Fig. 1d), resulting in a rotation of the target qubit conditional on the state of the control qubit. First, we initialize $\left\vert {{{\rm{Q1}}}},{{{\rm{Q2}}}}\right\rangle$ in the $\left\vert \downarrow \uparrow \right\rangle$-state by pulsing from *ϵ* > 0, where the spin-blockaded $\left\vert \downarrow \downarrow \right\rangle$-state is occupied, to *ϵ* = −2.9 meV, where *J*_∥_/*h* ≈ 80 MHz and MW-induced state leakage is suppressed^29^ (Supplementary Section 4). Subsequently, the state of the control qubit Q2 is prepared by a MW burst of length *t*_b2_ and frequency *f*_2↓_, and finally a CROT of the target qubit Q1 is triggered by the subsequent pulse with *t*_b1_ and *f*_1↑_ (Fig. 4a). The measurement outcome is presented in Fig. 4b, revealing the characteristic fading in and out of the target qubit’s Rabi oscillations as a function of *t*_b2_, that is, the spin state of the control qubit^5,35^. A controlled spin-flip for Q1 is executed in ~24 ns, which is short compared to other realizations with electrons in Si (ref. ^34^) or holes in Ge (refs. ^5,35^). We remark that our transport-based readout scheme prevents single-shot spin measurements and severely limits the duration of the qubits’ manipulation stage^22^, such that randomized benchmarking to determine a two-qubit gate fidelity could not be performed^49^.

A conditional spin-flip provides a natural way of implementing a controlled-NOT (CNOT) gate, differing from a CROT only by a phase factor. Two key requirements need to be fulfilled for high-fidelity CROT gates. First, to prevent a mixing of the antiparallel spin states ($\left\vert \uparrow \downarrow \right\rangle ,\left\vert \downarrow \uparrow \right\rangle$), the Zeeman energy difference between the qubits Δ*E*_Z_ must be much larger than the ‘perpendicular’ exchange coupling *J*_⊥_ (*J*_⊥_/*J*_∥_ induces SWAP/controlled-phase oscillations^29,30,40^). Second, either *J*_∥_ ≫ *hf*_Rabi_ or ${J}_{\parallel }/\sqrt{15}\,=\,h{f}_{{{{\rm{Rabi}}}}}$ to avoid unwanted rotations of the off-resonant states^34,40^. Hence, for electrons with isotropic exchange (*J*_∥_ = *J*_⊥_ = *J*) the speed of high-fidelity CROT gates is limited by *hf*_Rabi_ ≪ *J* ≪ Δ*E*_Z_. However, for hole spins with highly anisotropic exchange interaction, this limit can be overcome. In fact, *J*_∥_ = *J*_0_ while *J*_⊥_ = 0 is possible, for instance, if the *g*-tensors are isotropic, for *θ*_so_ = π/2 and **B** perpendicular to $\hat{n}_{\rm{so}}$ we remark that the latter condition also ensures fast single-qubit rotations. Consequently, our theory predicts that for holes in comparison to electrons, a CNOT gate with fidelity above 99% can be realized with much shorter gate times (Fig. 4c). The gate fidelities presented in Fig. 4c were numerically calculated in the absence of incoherent noise, that is, the gate infidelities are due to Hamiltonian errors^31^ (Supplementary Section 7). For the controlled rotation operation presented in Fig. 4b the magnetic field orientation (marked by the vertical orange line in Fig. 3b) was chosen such that both a close-to-ideal exchange configuration (∣*J*_∥_∣ = 0.90 *J*_0_, ∣*J*_⊥_∣ = 0.05 *J*_0_) and good readout contrast were achieved. In Fig. 3a–e the red dashed curves show the dependence of *J*_⊥_ on **B**/∣**B**∣, highlighting that the ideal configuration (*J*_∥_ ≈ *J*_0_, *J*_⊥_ ≈ 0) is stretched over a wide range of directions. The CROT sweet spot is consequently tolerant to device variations, making this concept suitable for large qubit arrays, a point reinforced by the low variability and disorder resulting from industrial manufacturing^21,50^ and the electrical tunability of the SOI^11,13^.

In summary, we investigated the exchange coupling between two hole-spins in a Si FinFET and found it to be both highly anisotropic and tunable, allowing for an interaction strength >0.5 GHz. We identify the strong SOI as the main microscopic origin of this anisotropy and propose a simple procedure for determining the exchange matrix. This measurement and analysis scheme applies to a wide variety of devices, for instance, to electron spin qubits with synthetic SOI in the presence of a magnetic field gradient (Supplementary Section 6)^4,29,34^. By fully characterizing the Hamiltonian of the two coupled spins, the best possible configuration for implementing two-qubit gates can be identified. A strongly anisotropic exchange results in extended sweet spots in magnetic field orientation, where both fast and high-fidelity CROTs can be performed. Finally, by choosing a close-to-ideal configuration we realize a controlled spin-flip in just ~24 ns.

Future improvements in device fabrication^21,50^, assisted by high-volume characterization^51,52^, are needed to reduce device variability. Low-variability devices, combined with robust CROT sweet spots, will make two-qubit gate operations with anisotropic exchange highly attractive for large-scale qubit arrays. The concepts presented here are, in principle, compatible with elevated temperatures, but experimental confirmation is presently lacking. The advances reported here, if they can be combined with fast readout^53^ and operation above 1 K, would show that industrial FinFET technology has great potential for realizing a universal quantum processor, integrated on the same chip with the classical control electronics.

## Methods

### Device fabrication

The fin structures are orientated along the [110] crystal direction on a near-intrinsic, natural Si substrate (*ρ* > 10 kΩ cm and (100) surface) and are covered by an ≃7-nm-thick, thermally grown silicon dioxide (SiO_2_) layer. Two layers of titanium nitride gate electrodes, which are electrically isolated by a ≃4.5-nm-thick SiO_2_ layer deposited by atomic layer deposition, are used for DQD formation. The second gate layer is integrated by a self-aligned process, resulting in a perfect layer-to-layer alignment. The p-type source and drain regions are made of platinum silicide. Finally, the devices are embedded in an ≃100-nm-thick SiO_2_ layer and are measured through contact vias filled with tungsten. Further details on the device fabrication are provided in refs. ^19,20^.

### Experimental setup

All measurements are performed using a Bluefors dry dilution refrigerator with a base temperature of ~40 mK and a three-axis magnet that provides arbitrary control of the magnetic field vector **B**. The d.c. voltages are supplied by a low-noise voltage source (BasPI SP927) and the fast pulses applied to the P1-gate (Fig. 1a) by an arbitrary waveform generator (Tektronix AWG5208), which also controls the I and Q inputs of a vector signal generator (Rohde & Schwarz SGS100A) for generating sideband-modulated EDSR microwave pulses. The source-to-drain current is measured with a current-to-voltage amplifier (BasPI SP983c) and a lock-in amplifier (Signal Recovery 7265), chopping the microwave signal at a frequency of 89.2 Hz for better noise rejection. Further details are provided in Supplementary Section 1.

### Derivation of the fit function for the exchange matrix

Using a Fermi–Hubbard model with a single orbital state $\left\vert i\right\rangle$ per site *i* = {1, 2}, our DQD system is described by the Hamiltonian

5 $${H}_{{{{\rm{FH}}}}}=\mathop{\sum}\limits_{i,\;j\in \{1,2\}}\mathop{\sum}\limits_{s{s}^{{\prime} }\in \{\uparrow ,\downarrow \}}{\tilde{H}}_{ij}^{s{s}^{{\prime} }}{a}_{is}^{{\dagger} }{a}_{j{s}^{{\prime} }}+U\mathop{\sum}\limits_{i\in \{1,2\}}{n}_{i\uparrow }{n}_{i\downarrow }.$$

Here ${a}_{is}^{{\dagger} }$ (*a*_*is*_) creates (removes) a hole on site *i* and spin $s=\{\left\vert \uparrow \right\rangle ,\left\vert \downarrow \right\rangle \},$ ${n}_{is}={a}_{is}^{{\dagger} }{a}_{is}$ is the occupation number operator, and *U* is the charging energy. The single-particle Hamiltonian $\tilde{H}$ is given by

6 $$\begin{array}{l}\tilde{H}=\frac{\tilde{\epsilon }}{2}{\tau }_{z}+{t}_{\rm{c}}\cos ({\theta }_{{{{\rm{so}}}}}){\tau }_{x}+{t}_{\rm{c}}\sin ({\theta }_{{{{\rm{so}}}}}){\tau }_{y}{{{{\bf{n}}}}}_{{{{\rm{so}}}}}\cdot {{{\mathbf{\upsigma }}}}+\frac{1}{2}{\mu }_{\rm{B}}{{{\bf{B}}}}\cdot \left[\frac{1+{\tau }_{z}}{2}{\hat{g}}_{1}\cdot {{{\mathbf{\upsigma }}}}\right.\\\left.\qquad+\frac{1-{\tau }_{z}}{2}{\hat{g}}_{2}\cdot {{{\mathbf{\upsigma }}}}\right],\end{array}$$

and contains spin-conserving interdot tunnelling ${t}_{\rm{c}}\cos ({\theta }_{{{{\rm{so}}}}}){\tau }_{x}$ and an SOI-induced spin-flip hopping term ${t}_{\rm{c}}\sin ({\theta }_{{{{\rm{so}}}}}){\tau }_{y}{{{{\hat{\bf{n}}}}}}_{{{{\rm{so}}}}}\cdot {{{\mathbf{\upsigma }}}}$. Here we use the convention that the gap size of the anticrossing of two tunnel-coupled states is given by $2\sqrt{2}{t}_{\rm{c}}$. Moreover, (*τ*_*x*_, *τ*_*y*_, *τ*_*z*_) are the Pauli matrices for the orbital degree of freedom, for example ${\tau }_{z}=\left\vert 1\right\rangle \left\langle 1\right\vert -\left\vert 2\right\rangle \left\langle 2\right\vert$, and **σ** is the vector of Pauli matrices acting on the spin degree of freedom. In the laboratory frame, as defined in Fig. 1, the *g*-tensors ${\hat{g}}_{1}$ and ${\hat{g}}_{2}$ are symmetric (Supplementary Section 3). Finally, $\tilde{\epsilon }$ is the energy difference for a hole occupying the left or the right QD and is expressed in terms of the detuning energy *ϵ* between the (1,1) and (0,2) charge states by $\tilde{\epsilon }=\epsilon +U-{U}_{0}$.

We perform a transformation from the laboratory frame to the so-called ‘spin–orbit frame’ and find

7 $${\tilde{H}}^{{{{\rm{so}}}}}={U}_{{{{\rm{so}}}}}^{{\dagger} }\tilde{H}{U}_{{{{\rm{so}}}}}=\frac{\tilde{\epsilon }}{2}{\tau }_{z}+{t}_{\rm{c}}{\tau }_{x}+\frac{1}{2}{\mu }_{\rm{B}}{{{\bf{B}}}}\cdot \left[\frac{1+{\tau }_{z}}{2}{\hat{g}}_{1}^{{{{\rm{so}}}}}\cdot {{{\mathbf{\upsigma }}}}+\frac{1-{\tau }_{z}}{2}{\hat{g}}_{2}^{{{{\rm{so}}}}}\cdot {{{\mathbf{\upsigma }}}}\right].$$

In the spin–orbit frame, non-spin-conserving tunnelling is gauged away by the unitary transformation ${U}_{{{{\rm{so}}}}}=\exp (-i{\theta }_{{{{\rm{so}}}}}{\tau }_{z}{{{{\hat{\bf{n}}}}}}_{{{{\rm{so}}}}}\cdot {{{\mathbf{\upsigma }}}}/2)$, and the *g*-tensors are given by ${\hat{g}}_{1}^{\,{{{\rm{so}}}}}={\hat{g}}_{1}\cdot {\hat{R}}_{{{{\rm{so}}}}}({\theta }_{{{{\rm{so}}}}})$ and ${\hat{g}}_{2}^{{{{\rm{so}}}}}={\hat{g}}_{2}\cdot {\hat{R}}_{{{{\rm{so}}}}}(-{\theta }_{{{{\rm{so}}}}})$. Here ${\hat{R}}_{{{{\rm{so}}}}}(\varphi )$ denotes a counterclockwise rotation around $\hat{\bf{n}}_{\rm{so}}$ by an angle *φ*. As our DQD system is operated close to the $\left\vert {S}_{02}\right\rangle$-$\left\vert S\right\rangle$ anticrossing, the Hamiltonian *H*_FH_ can be represented in the basis $\{\left\vert {S}_{02}\right\rangle ,\left\vert S\right\rangle ,\left\vert {T}_{-}\right\rangle ,\left\vert {T}_{+}\right\rangle ,\left\vert {T}_{0}\right\rangle \}$

8 $${H}_{5\times 5}=\left(\begin{array}{ccccc}{U}_{0}-\epsilon &\sqrt{2}{t}_{{\rm{c}}}&0&0&0\\ \sqrt{2}{t}_{\rm{c}}&0&-\frac{\delta {{{{\rm{b}}}}}_{x}+i\delta {{{{\rm{b}}}}}_{y}}{\sqrt{2}}&\frac{\delta {{{{\rm{b}}}}}_{x}-i\delta {{{{\rm{b}}}}}_{y}}{\sqrt{2}}&\delta {{{{\rm{b}}}}}_{z}\\ 0&-\frac{\delta {{{{\rm{b}}}}}_{x}-i\delta {{{{\rm{b}}}}}_{y}}{\sqrt{2}}&{\bar{b}}_{z}&0&\frac{{\bar{b}}_{x}-i{\bar{b}}_{y}}{\sqrt{2}}\\ 0&\frac{\delta {{{{\rm{b}}}}}_{x}+i\delta {{{{\rm{b}}}}}_{y}}{\sqrt{2}}&0&-{\bar{b}}_{z}&\frac{{\bar{b}}_{x}+i{\bar{b}}_{y}}{\sqrt{2}}\\ 0&\delta {{{{\rm{b}}}}}_{z}&\frac{{\bar{b}}_{x}+i{\bar{b}}_{y}}{\sqrt{2}}&\frac{{\bar{b}}_{x}-i{\bar{b}}_{y}}{\sqrt{2}}&0\end{array}\right),$$

where the average and gradient Zeeman fields $\bar{b}={\mu }_{\rm{B}}{{{\bf{B}}}}\cdot ({\hat{g}}_{1}^{{{{\rm{so}}}}}+{\hat{g}}_{2}^{{{{\rm{so}}}}})/2$ and $\delta {{{\rm{b}}}}={\mu }_{\rm{B}}{{{\bf{B}}}}\cdot ({\hat{g}}_{1}^{{{{\rm{so}}}}}-{\hat{g}}_{2}^{{{{\rm{so}}}}})/2$ were introduced. In the spin–orbit frame, the singlet subspace $\{\left\vert {S}_{02}\right\rangle ,\left\vert S\right\rangle \}$ is coupled by the total tunnel coupling *t*_c_ and the hybridized singlets *S*_±_ have energies ${E}_{{S}_{+}}={U}_{0}-\epsilon +{J}_{0}$ and ${E}_{{S}_{-}}=-{J}_{0}$ with ${J}_{0}=\sqrt{2}\tan (\gamma /2)=-({U}_{0}-\epsilon )[1-\sqrt{1+8{t}_{\rm{c}}^{2}/{({U}_{0}-\epsilon )}^{2}}]/2$ and mixing angle $\gamma =\arctan [\sqrt{8}{t}_{\rm{c}}/({U}_{0}-\epsilon )]$. Furthermore, we remark that ${J}_{0}\approx 2{t}_{\rm{c}}^{2}/({U}_{0}-\epsilon )$ in the limit of *t*_c_/(*U*_0_ − *ϵ*) ≪ 1. Because *S*_+_ couples only weakly to the triplet states, our Hilbert space can be restricted to the four levels $\{\left\vert {S}_{-}\right\rangle ,\left\vert {T}_{-}\right\rangle ,\left\vert {T}_{+}\right\rangle ,\left\vert {T}_{0}\right\rangle \}$ and we obtain

9 $${H}_{4\times 4}=\left(\begin{array}{cccc}-{J}_{0}&-\frac{\delta {{{{\rm{b}}}}}_{x}+i\delta {{{{\rm{b}}}}}_{y}}{\sqrt{2}}\cos \left(\frac{\gamma }{2}\right)&\frac{\delta {{{{\rm{b}}}}}_{x}-i\delta {{{{\rm{b}}}}}_{y}}{\sqrt{2}}\cos \left(\frac{\gamma }{2}\right)&\delta {{{{\rm{b}}}}}_{z}\cos \left(\frac{\gamma }{2}\right)\\ -\frac{\delta {{{{\rm{b}}}}}_{x}-i\delta {{{{\rm{b}}}}}_{y}}{\sqrt{2}}\cos \left(\frac{\gamma }{2}\right)&{\bar{b}}_{z}&0&\frac{{\bar{b}}_{x}-i{\bar{b}}_{y}}{\sqrt{2}}\\ \frac{\delta {{{{\rm{b}}}}}_{x}+i\delta {{{{\rm{b}}}}}_{y}}{\sqrt{2}}\cos \left(\frac{\gamma }{2}\right)&0&-{\bar{b}}_{z}&\frac{{\bar{b}}_{x}+i{\bar{b}}_{y}}{\sqrt{2}}\\ \delta {{{{\rm{b}}}}}_{z}\cos \left(\frac{\gamma }{2}\right)&\frac{{\bar{b}}_{x}+i{\bar{b}}_{y}}{\sqrt{2}}&\frac{{\bar{b}}_{x}-i{\bar{b}}_{y}}{\sqrt{2}}&0\end{array}\right).$$

Hole spin manipulation is performed deep in the (1,1) charge stability region, allowing us to introduce the localized spin operators ${{{{\mathbf{\upsigma}}}}}_{1}^{{{{\rm{so}}}}}$ and ${{{{\mathbf{\upsigma}}}}}_{2}^{{{{\rm{so}}}}}$. The Hamiltonian (9) can then be written as

10 $${H}_{(1,1)}^{{{{\rm{so}}}}}=\frac{1}{2}{\mu }_{\rm{B}}{{{\bf{B}}}}\cdot {\hat{g}}_{1}^{{{{\rm{so}}}}}\cdot {{{{\mathbf{\upsigma }}}}}_{1}^{{{{\rm{so}}}}}+\frac{1}{2}{\mu }_{\rm{B}}{{{\bf{B}}}}\cdot {\hat{g}}_{2}^{{{{\rm{so}}}}}\cdot {{{{\mathbf{\upsigma }}}}}_{2}^{{{{\rm{so}}}}}+\frac{1}{4}{J}_{0}{{{{\mathbf{\upsigma }}}}}_{1}^{{{{\rm{so}}}}}\cdot {{{{\mathbf{\upsigma }}}}}_{2}^{{{{\rm{so}}}}}\,,$$

revealing that the exchange interaction is isotropic in the spin–orbit frame. To find an expression for the experimentally measured values, we first rewrite equation (10) in the lab frame:

11 $${H}_{(1,1)}^{\,{{{\rm{lab}}}}}=\frac{1}{2}{\mu }_{\rm{B}}{{{\bf{B}}}}\cdot {\hat{g}}_{1}\cdot {{{{\mathbf{\upsigma }}}}}_{1}+\frac{1}{2}{\mu }_{\rm{B}}{{{\bf{B}}}}\cdot {\hat{g}}_{2}\cdot {{{{\mathbf{\upsigma }}}}}_{2}+\frac{1}{4}{{{{\mathbf{\upsigma }}}}}_{1}\cdot \hat{{{{\mathcal{J}}}}}\cdot {{{{\mathbf{\upsigma }}}}}_{2}\,.$$

Here $\hat{{{{\mathcal{J}}}}}={J}_{0}{\hat{R}}_{{{{\rm{so}}}}}(-2{\theta }_{{{{\rm{so}}}}})$ represents the exchange matrix in the lab frame, ${{{{\mathbf{\upsigma }}}}}_{1}={\hat{R}}_{{{{\rm{so}}}}}(-{\theta }_{{{{\rm{so}}}}})\cdot {{{{\mathbf{\upsigma }}}}}_{1}^{{{{\rm{so}}}}}$ and ${{{{\mathbf{\upsigma }}}}}_{2}={\hat{R}}_{{{{\rm{so}}}}}({\theta }_{{{{\rm{so}}}}})\cdot {{{{\mathbf{\upsigma }}}}}_{2}^{{{{\rm{so}}}}}$. In addition, independent rotations ${\hat{R}}_{1}$ and ${\hat{R}}_{2}$ are applied to Q1 and Q2, such that the single-particle terms of the Hamiltonian (11) become diagonal:

12 $${H}_{(1,1)}^{\,{{{\rm{Q}}}}}=\frac{1}{2}{E}_{Z,1}{\sigma }_{z,1}^{{{{\rm{Q}}}}}+\frac{1}{2}{E}_{Z,2}{\sigma }_{z,2}^{{{{\rm{Q}}}}}+\frac{1}{4}{{{{\mathbf{\upsigma }}}}}_{1}^{{{{\rm{Q}}}}}\cdot {\hat{{{{\mathcal{J}}}}}}^{{{{\rm{Q}}}}}\cdot {{{{\mathbf{\upsigma }}}}}_{2}^{{{{\rm{Q}}}}}\,,$$

where ${E}_{Z,i}{{{{\bf{e}}}}}_{z}^{{{{\rm{Q}}}}}={\mu }_{\rm{B}}{\hat{R}}_{i}\cdot {\hat{g}}_{i}\cdot {{{\bf{B}}}}$ is the *i*-th site’s Zeeman splitting, ${{{{\bf{e}}}}}_{z}^{{{{\rm{Q}}}}}$ the spin quantization axis and ${\hat{{{{\mathcal{J}}}}}}^{{{{\rm{Q}}}}}={J}_{0}{\hat{R}}_{1}\cdot {\hat{R}}_{{{{\rm{so}}}}}(-2{\theta }_{{{{\rm{so}}}}})\cdot {\hat{R}}_{2}^{T}$ the exchange matrix in the so-called ‘qubit frame’, wherein the exchange splitting *J*_∥_ is experimentally observed. To obtain an expression for *J*_∥_ we rewrite the Hamiltonian of equation (12) in matrix form using the two-qubit basis $\{\left\vert \uparrow \uparrow \right\rangle ,\left\vert \uparrow \downarrow \right\rangle ,\left\vert \downarrow \uparrow \right\rangle ,\left\vert \downarrow \downarrow \right\rangle \}$

13 $${H}_{(1,1)}^{\,{{{\rm{Q}}}}}=\left(\begin{array}{cccc}{E}_{Z}+\frac{1}{4}{J}_{zz}^{\,{{{\rm{Q}}}}}&0&0&0\\ 0&\frac{1}{2}\Delta {E}_{Z}-\frac{1}{4}{J}_{zz}^{\,{{{\rm{Q}}}}}&\frac{1}{2}{J}_{\perp }&0\\ 0&\frac{1}{2}{(\;{J}_{\perp })}^{* }&-\frac{1}{2}\Delta {E}_{Z}-\frac{1}{4}{J}_{zz}^{\,{{{\rm{Q}}}}}&0\\ 0&0&0&-{E}_{Z}+\frac{1}{4}{J}_{zz}^{\,{{{\rm{Q}}}}}\end{array}\right).$$

Here we neglect every coupling that would contribute to the eigenvalues in ${{{\mathcal{O}}}}(\;{J}_{0}^{2}/{E}_{Z})$ and introduce ${J}_{\perp }=[\;{J}_{xx}^{\,{{{\rm{Q}}}}}+{J}_{yy}^{\,{{{\rm{Q}}}}}+i(\,{J}_{xy}^{\,{{{\rm{Q}}}}}-{J}_{yx}^{\,{{{\rm{Q}}}}})]/2,{E}_{Z}=\left({E}_{Z,1}\right.$ $\left.+{E}_{Z,2}\right)/2$ and Δ*E*_*Z*_ = *E*_*Z*,1_ − *E*_*Z*,2_. The eigenenergies of equation (13) are

14a $${E}_{\uparrow \uparrow }={E}_{Z}+\frac{1}{4}{J}_{zz}^{\,{{{\rm{Q}}}}},\quad {E}_{\downarrow \downarrow }=-{E}_{Z}+\frac{1}{4}{J}_{zz}^{\,{{{\rm{Q}}}}},$$

14b $${E}_{\widetilde{\uparrow \downarrow }}=\frac{1}{2}\Delta {\tilde{E}}_{Z}-\frac{1}{4}{J}_{zz}^{\,{{{\rm{Q}}}}},\quad {E}_{\widetilde{\downarrow \uparrow }}=-\frac{1}{2}\Delta {\tilde{E}}_{Z}-\frac{1}{4}{J}_{zz}^{\,{{{\rm{Q}}}}},$$

with $\Delta {\tilde{E}}_{Z}=\sqrt{\Delta {E}_{Z}^{2}+|\, {J}_{\perp }{| }^{2}}$. We thus find for the exchange splitting, which is defined as the energy difference between the two transitions flipping the same spin, ${J}_{\parallel }={E}_{\uparrow \uparrow }-{E}_{\widetilde{\uparrow \downarrow }}-({E}_{\widetilde{\downarrow \uparrow }}-{E}_{\downarrow \downarrow })={J}_{zz}^{\,{{{\rm{Q}}}}}$. The matrix element ${J}_{zz}^{\,{{{\rm{Q}}}}}$ is in turn given by

15 $${J}_{zz}^{\,{{{\rm{Q}}}}}={J}_{\parallel }={{{{\bf{e}}}}}_{z}^{{{{\rm{Q}}}}}\cdot {\hat{{{{\mathcal{J}}}}}}^{{{{\rm{Q}}}}}\cdot {{{{\bf{e}}}}}_{z}^{{{{\rm{Q}}}}}={{{{\hat{\bf{n}}}}}}_{1}\cdot \hat{{{{\mathcal{J}}}}}\cdot {{{{\hat{\bf{n}}}}}}_{2}={J}_{0}\,{{{{\hat{\bf{n}}}}}}_{1}\cdot {\hat{R}}_{{{{\rm{so}}}}}(-2{\theta }_{{{{\rm{so}}}}})\cdot {{{{\hat{\bf{n}}}}}}_{2}\,.$$

equation (15) is the fit function employed to describe the observed exchange anisotropy, where the effect of both SOI and the anisotropy of the *g*-tensors is accounted for. We note that an explicit dependence on the magnetic field direction arises from ${{{{\hat{\bf{n}}}}}_{i}={\hat{g}}_{i}\cdot {{{\bf{B}}}}/| {\hat{g}}_{i}\cdot {{{\bf{B}}}}|}$. Further details of the derivation are found in Supplementary Section 5.

### Numerical calculation of the CNOT gate fidelity

The CROT gate operation is modelled by numerically evaluating the Hamiltonian’s time evolution

16 $$\mathrm{CROT}_{\mathrm{num}}=\mathcal{T} \exp \left[ -\frac{i}{\hbar}\int_0^{t_{\uppi}}{}\!H_{(1,1)}^{\,\mathrm{Q}}(t)\mathrm{d}t \right] .$$

Here ${{{\mathcal{T}}}}$ denotes time-ordering, *t*_π_ is the spin-flip time, and the time-dependent Hamiltonian ${H}_{(1,1)}^{\,{{{\rm{Q}}}}}(t)$ results from equation (13) after adding the drive $h{f}_{{{{\rm{Rabi}}}}}\,\sin (2\uppi {f}_{1\uparrow }\,t)\,{\sigma }_{x,1}$, where the Rabi frequency fulfils the condition $h{f}_{{{{\rm{Rabi}}}}}={J}_{\parallel }/\sqrt{15}$ to suppress off-resonant driving^34,40^. Finally, the CNOT gate fidelity is determined by ${{{\mathcal{F}}}}=\frac{1}{4}| \,{{\mbox{Tr}}}\,$ $\left[{{{\mbox{CNOT}}}}_{{{{\rm{num}}}}}\,{{{\mbox{CNOT}}}}^{{\dagger} }\right]|$ where CNOT is the ideal gate matrix and CNOT_num_ is obtained by applying single-qubit phase corrections to equation (16). For more details, see Supplementary Section 7.

## Online content

Any methods, additional references, Nature Portfolio reporting summaries, source data, extended data, supplementary information, acknowledgements, peer review information; details of author contributions and competing interests; and statements of data and code availability are available at 10.1038/s41567-024-02481-5.

## Supplementary information

Supplementary Information Supplementary Sections 1–8 and Figs. 1–6.

## Source data

Source Data Fig. 1 All data shown in Fig. 1.

Source Data Fig. 2 All data shown in Fig. 2.

Source Data Fig. 3 All data shown in Fig. 3.

Source Data Fig. 4 All data shown in Fig. 4.

## Data Availability

The data supporting the plots within this paper are available via Zenodo at 10.5281/zenodo.7547764 (ref. ^54^). Source data are provided with this paper.

## References

[1] Loss, D. & DiVincenzo, D. P. Quantum computation with quantum dots. *Phys. Rev. A* **57**, 120–126 (1998).

[2] Vandersypen, L. M. K. et al. Interfacing spin qubits in quantum dots and donors-hot, dense, and coherent. *npj Quantum Inf* **3**, 34 (2017).

[3] Veldhorst, M., Eenink, H. G. J., Yang, C. H. & Dzurak, A. S. Silicon CMOS architecture for a spin-based quantum computer. *Nat. Commun.* **8**, 1766 (2017). 29242497 10.1038/s41467-017-01905-6 PMC5730618

[4] Philips, S. G. J. et al. Universal control of a six-qubit quantum processor in silicon. *Nature* **609**, 919–924 (2022). 36171383 10.1038/s41586-022-05117-x PMC9519456

[5] Hendrickx, N. W. et al. A four-qubit germanium quantum processor. *Nature* **591**, 580–585 (2021). 33762771 10.1038/s41586-021-03332-6

[6] Tokura, Y., van der Wiel, W. G., Obata, T. & Tarucha, S. Coherent single electron spin control in a slanting Zeeman field. *Phys. Rev. Lett.* **96**, 047202 (2006). 16486882 10.1103/PhysRevLett.96.047202

[7] Pioro-Ladrière, M. et al. Electrically driven single-electron spin resonance in a slanting Zeeman field. *Nat. Phys.* **4**, 776–779 (2008).

[8] Gilbert, W. et al. On-demand electrical control of spin qubits. *Nat. Nanotechnol.* **18**, 131–136 (2023). 36635331 10.1038/s41565-022-01280-4

[9] Prechtel, J. H. et al. Decoupling a hole spin qubit from the nuclear spins. *Nat. Mater.* **15**, 981–986 (2016). 27454044 10.1038/nmat4704

[10] Zwanenburg, F. A. et al. Silicon quantum electronics. *Rev. Mod. Phys.* **85**, 961 (2013).

[11] Kloeffel, C., Rančić, M. J. & Loss, D. Direct Rashba spin-orbit interaction in Si and Ge nanowires with different growth directions. *Phys. Rev. B* **97**, 235422 (2018).

[12] Bosco, S. & Loss, D. Fully tunable hyperfine interactions of hole spin qubits in Si and Ge quantum dots. *Phys. Rev. Lett.* **127**, 190501 (2021). 34797148 10.1103/PhysRevLett.127.190501

[13] Bosco, S., Hetényi, B. & Loss, D. Hole spin qubits in Si FinFETs with fully tunable spin-orbit coupling and sweet spots for charge noise. *PRX Quantum* **2**, 010348 (2021).

[14] Froning, F. N. M. et al. Ultrafast hole spin qubit with gate-tunable spin–orbit switch functionality. *Nat. Nanotechnol.* **16**, 308–312 (2021). 33432204 10.1038/s41565-020-00828-6

[15] Wang, K. et al. Ultrafast coherent control of a hole spin qubit in a germanium quantum dot. *Nat. Commun.* **13**, 206 (2022). 35017522 10.1038/s41467-021-27880-7 PMC8752786

[16] Piot, N. et al. A single hole spin with enhanced coherence in natural silicon. *Nat. Nanotechnol.* **17**, 1072–1077 (2022). 36138200 10.1038/s41565-022-01196-z PMC9576591

[17] Auth, C. et al. A 22nm high performance and low-power CMOS technology featuring fully-depleted tri-gate transistors, self-aligned contacts and high density MIM capacitors. In *2012 Symposium on VLSI Technology (VLSIT)* 131–132 (IEEE, 2012).

[18] Maurand, R. et al. A CMOS silicon spin qubit. *Nat. Commun.* **7**, 13575 (2016). 27882926 10.1038/ncomms13575 PMC5123048

[19] Kuhlmann, A. V., Deshpande, V., Camenzind, L. C., Zumbühl, D. M. & Fuhrer, A. Ambipolar quantum dots in undoped silicon fin field-effect transistors. *Appl. Phys. Lett.* **113**, 122107 (2018).

[20] Geyer, S. et al. Self-aligned gates for scalable silicon quantum computing. *Appl. Phys. Lett.* **118**, 104004 (2021).

[21] Zwerver, A. M. J. et al. Qubits made by advanced semiconductor manufacturing. *Nat. Electron.* **5**, 184–190 (2022).

[22] Camenzind, L. C. et al. A hole spin qubit in a fin field-effect transistor above 4 kelvin. *Nat. Electron.* **5**, 178–183 (2022).

[23] Gonzalez-Zalba, M. F. et al. Scaling silicon-based quantum computing using CMOS technology. *Nat. Electron.* **4**, 872–884 (2021).

[24] Petit, L. et al. Universal quantum logic in hot silicon qubits. *Nature* **580**, 355–359 (2020). 32296188 10.1038/s41586-020-2170-7

[25] Yang, C. H. et al. Operation of a silicon quantum processor unit cell above one kelvin. *Nature* **580**, 350–354 (2020). 32296190 10.1038/s41586-020-2171-6

[26] Xue, X. et al. CMOS-based cryogenic control of silicon quantum circuits. *Nature* **593**, 205–210 (2021). 33981049 10.1038/s41586-021-03469-4

[27] Petta, J. R. et al. Coherent manipulation of coupled electron spins in semiconductor quantum dots. *Science* **309**, 2180–2184 (2005). 16141370 10.1126/science.1116955

[28] Veldhorst, M. et al. A two-qubit logic gate in silicon. *Nature* **526**, 410–414 (2015). 26436453 10.1038/nature15263

[29] Watson, T. F. et al. A programmable two-qubit quantum processor in silicon. *Nature* **555**, 633–637 (2018). 29443962 10.1038/nature25766

[30] Mills, A. R. et al. Two-qubit silicon quantum processor with operation fidelity exceeding 99%. *Sci. Adv.* **8**, 14 (2022). 10.1126/sciadv.abn5130 PMC8986105 35385308

[31] Xue, X. et al. Quantum logic with spin qubits crossing the surface code threshold. *Nature* **601**, 343–347 (2022). 35046604 10.1038/s41586-021-04273-w PMC8770146

[32] Zajac, D. M. et al. Resonantly driven CNOT gate for electron spins. *Science* **359**, 439–442 (2018). 29217586 10.1126/science.aao5965

[33] Huang, W. et al. Fidelity benchmarks for two-qubit gates in silicon. *Nature* **569**, 532–536 (2019). 31086337 10.1038/s41586-019-1197-0

[34] Noiri, A. et al. Fast universal quantum gate above the fault-tolerance threshold in silicon. *Nature* **601**, 338–342 (2022). 35046603 10.1038/s41586-021-04182-y

[35] Hendrickx, N. W. et al. A single-hole spin qubit. *Nat. Commun.* **11**, 3478 (2020). 32651363 10.1038/s41467-020-17211-7 PMC7351715

[36] Fang, Y., Philippopoulos, P., Culcer, D., Coish, W. A. & Chesi, S. Recent advances in hole-spin qubits. *Mater. Quantum Technol.* **3**, 012003 (2023).

[37] Golovach, V. N., Borhani, M. & Loss, D. Electric-dipole-induced spin resonance in quantum dots. *Phys. Rev. B* **74**, 165319 (2006).

[38] Nowack, K. C., Koppens, F. H. L., Nazarov, Y. V. & Vandersypen, L. M. K. Coherent control of a single electron spin with electric fields. *Science* **318**, 1430–1433 (2007). 17975030 10.1126/science.1148092

[39] Stepanenko, D., Rudner, M., Halperin, B. I. & Loss, D. Singlet-triplet splitting in double quantum dots due to spin-orbit and hyperfine interactions. *Phys. Rev. B* **85**, 075416 (2012).

[40] Russ, M. et al. High-fidelity quantum gates in Si/SiGe double quantum dots. *Phys. Rev. B* **97**, 085421 (2018).

[41] Hendrickx, N. W., Franke, D. P., Sammak, A., Scappucci, G. & Veldhorst, M. Fast two-qubit logic with holes in germanium. *Nature* **577**, 487–491 (2020). 31932731 10.1038/s41586-019-1919-3

[42] Nadj-Perge, S. et al. Spectroscopy of spin-orbit quantum bits in indium antimonide nanowires. *Phys. Rev. Lett.* **108**, 166801 (2012). 22680747 10.1103/PhysRevLett.108.166801

[43] Froning, F. N. M. et al. Strong spin-orbit interaction and *g*-factor renormalization of hole spins in Ge/Si nanowire quantum dots. *Phys. Rev. Res.* **3**, 013081 (2021).

[44] Kavokin, K. V. Anisotropic exchange interaction of localized conduction-band electrons in semiconductors. *Phys. Rev. B* **64**, 075305 (2001).

[45] Kavokin, K. V. Symmetry of anisotropic exchange interactions in semiconductor nanostructures. *Phys. Rev. B* **69**, 075302 (2004).

[46] Hetényi, B., Kloeffel, C. & Loss, D. Exchange interaction of hole-spin qubits in double quantum dots in highly anisotropic semiconductors. *Phys. Rev. Res.* **2**, 033036 (2020).

[47] Hetényi, B., Bosco, S. & Loss, D. Anomalous zero-field splitting for hole spin qubits in Si and Ge quantum dots. *Phys. Rev. Lett.* **129**, 116805 (2022). 36154408 10.1103/PhysRevLett.129.116805

[48] Katsaros, G. et al. Zero field splitting of heavy-hole states in quantum dots. *Nano Lett.* **20**, 5201–5206 (2020). 32479090 10.1021/acs.nanolett.0c01466 PMC7349564

[49] Knill, E. et al. Randomized benchmarking of quantum gates. *Phys. Rev. A* **77**, 012307 (2008).

[50] Elsayed, A. et al. Low charge noise quantum dots with industrial CMOS manufacturing. Preprint at 10.48550/arXiv.2212.06464 (2022).

[51] de Kruijf, M. et al. A compact and versatile cryogenic probe station for quantum device testing. *Rev. Sci. Instrum.* **94**, 054707, 10.1063/5.0139825 (2023). 37204282 10.1063/5.0139825

[52] Neyens, S. et al. Probing single electrons across 300 mm spin qubit wafers. Preprint at 10.48550/arXiv.2307.04812 (2023). 10.1038/s41586-024-07275-6 PMC11062914 38693414

[53] Huang, J. Y. et al. A high-sensitivity charge sensor for silicon qubits above 1 K. *Nano Lett.* **21**, 6328–6335 (2021). 33999635 10.1021/acs.nanolett.1c01003

[54] Geyer, S. & Kuhlmann, A. Supporting Data for “Anisotropic exchange interaction of two hole spin qubitsˮ. *Zenodo*10.5281/zenodo.7547764 (2024). 10.1038/s41567-024-02481-5 PMC11631753 39664598

