# Supplementary material for: Anisotropic exchange interaction of two hole-spin qubits
Source: Nat Phys. 2024 May 6;20(7):1152–7. doi: 10.1038/s41567-024-02481-5 (PMC11631753; doi:10.1038/s41567-024-02481-5)
Supplement: Supplementary file 1 — Supplementary Sections 1–8 and Figs. 1–6. [file 41567_2024_2481_MOESM1_ESM.pdf]

nature physics

Article

https://doi.org/10.1038/s41567-024-02481-5

Anisotropic exchange interaction of two hole-spin qubits

In the format provided by the authors and unedited

CONTENTS

S1. Setup 2

S2. Spectroscopy data for qubit anisotropy characterization 3

S3. g-tensors for Q1 and Q2 4

S4. Two-qubit initialization 5

S5. Derivation of exchange matrix formalism and fitting formula 6

S6. Exchange matrix for electron QDs in silicon 12

S7. Theoretical limit of the CNOT gate fidelity 13

S8. Additional information to Fig. 2 19

References 19

S1. SETUP

In this section we discuss the experimental setup as well as the qubit operation. A schematic of the setup is presented in Fig. S1a. We performed all experiments by measuring direct current through a hole double quantum dot (DQD) at the base temperature $\sim 40\,\mathrm{mK}$ of a Bluefors XLD dilution refrigerator. Spin-to-charge conversion and spin initialization were realized using Pauli spin blockade in parity readout mode, which has been shown to be compatible with temperatures as high as 4 K, albeit with a reduction in readout signal and limited manipulation time [1]. The pulse scheme for each experiment cycle is described in Fig. S1b. Coherent single-qubit spin driving is demonstrated by a Rabi chevron measurement, presented in Fig. S1d. A more detailed description is found in the Supplementary Material to Ref. [1]. A key difference to this previously reported setup is the use of side-band modulation (SB) in the amplitude-quadrature (IQ) mixing of the microwave signal, which allows to quickly address different qubit frequencies in a single experiment cycle, thus enabling two-qubit experiments. Furthermore, this setup hosts a three-axis vector magnet.

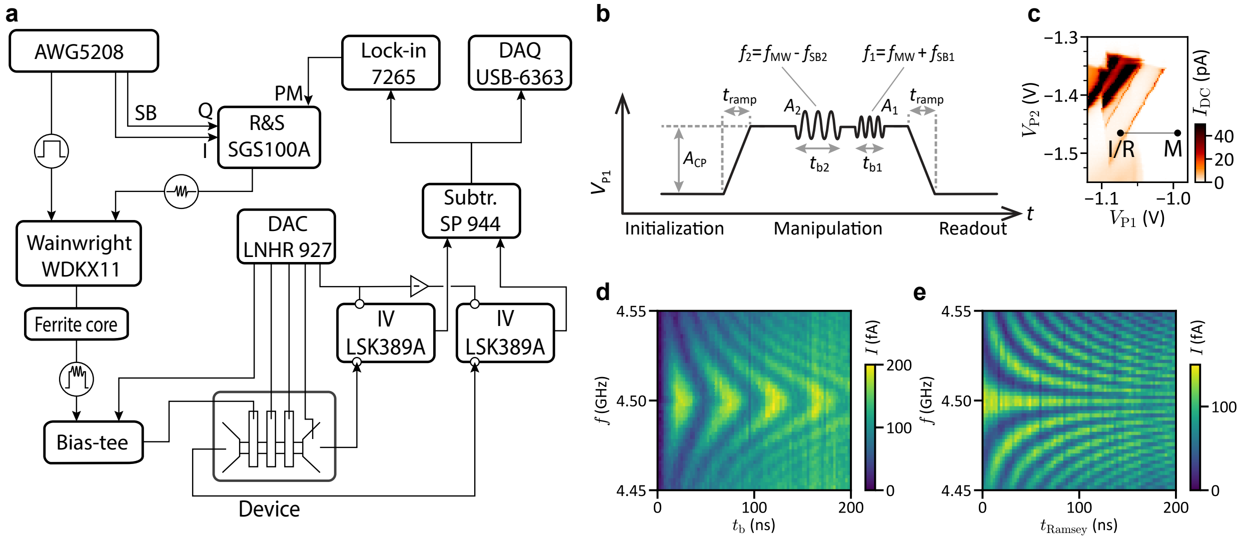

FIG. S1. **Setup and single qubit control.** **a**, Schematic of the experimental setup. The following instruments were used: a arbitrary waveform generator AWG5208 from Tektronix, a diplexer WDKX11+10-DC-1000/1300-15000-60S3 from Wainwright, a microwave signal generator SGS100A from Rhode&Schwarz, a lock-in amplifier Model 7265 DSP from Signal Recovery, a data acquisition card USB-6363 from National Instruments. Further, a voltage subtractor SP944, two current-voltage converters LSK389A and a digital-analogue converter LNHR927, all from Basel Precision Instruments, were used. **b**, Initialization, two-qubit manipulation and readout schematic. **c**, Charge stability diagram indicating initialization/readout and manipulation configuration. **d,e** Typical Rabi chevron and Ramsey fringe measurement of Q1.

S2. SPECTROSCOPY DATA FOR QUBIT ANISOTROPY CHARACTERIZATION

Here we present the raw data of the qubit spectroscopy experiments that were used to extract the g-tensors $\hat{g}_i$ of Q1 and Q2 and the exchange matrix $\hat{\mathcal{J}}$. Further, we observe correlations between the qubit readout signal in the lock-in current and the DC current through the base line of the bias triangle.

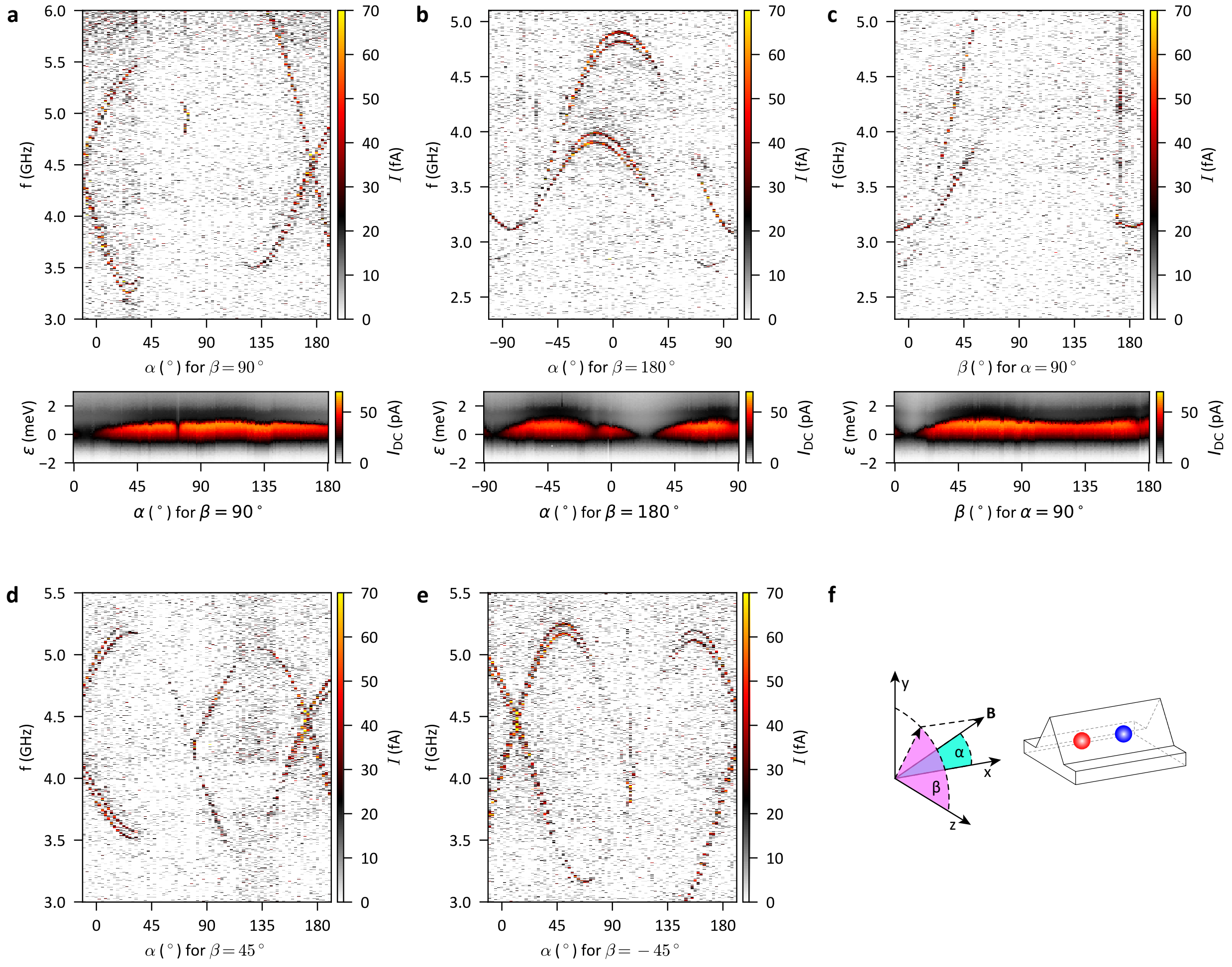

FIG. S2. **Qubit spectroscopy data.** **a-e**, Spectroscopy measurement as a function of magnetic field orientation (α, β) for sweeping $\mathbf{B}$ along 5 different planes with $V_\mathrm{B} = -820\,\mathrm{mV}$ and $\epsilon = -4.025\,\mathrm{meV}$. For a fixed magnetic field orientation 4 transitions can be identified as described in Fig. 1, which allows to extract $E_\mathrm{Z,i}$ and $J_{||}$ for each configuration. The gaps in the data come from a vanishing qubit readout signal for certain magnetic field orientations. Note that for some orientations only 1-3 transitions are vanishing. For **a-c** we additionally show the direct current I_DC of the zero detuning transition of the DQD as a function of magnetic field orientation at $|\mathbf{B}| = 0.1\,\mathrm{T}$. A correlation between a large current and a vanishing qubit visibility is observed. **f** Coordinate system and definition of the sweep parameters α and β.

S3. g-TENSORS FOR Q1 AND Q2

The g-tensors were extracted according to Ref. [2] by measuring $E_{\mathrm{Z,i}}$ by MW spectroscopy in at least 6 different orientations. The extraction was performed on the data presented in Fig. S2.

$$\hat{g}_1 = \begin{pmatrix} 2.31 & 0.50 & -0.06 \\ 0.50 & 2.00 & 0.06 \\ -0.06 & 0.06 & 1.50 \end{pmatrix}, \qquad \hat{g}_2 = \begin{pmatrix} 1.86 & -0.57 & 0.09 \\ -0.57 & 2.76 & -0.01 \\ 0.09 & -0.01 & 1.46 \end{pmatrix} \tag{1}$$

The g-tensors can be diagonalized, such that the effective g-factors along the principal axes can be easily read off:

$$\hat{g}_1^{\mathrm{diag}} = \mathrm{diag}\,(2.68, 1.68, 1.46)\,, \qquad \hat{g}_2^{\mathrm{diag}} = \mathrm{diag}\,(3.04, 1.62, 1.42)\,. \tag{2}$$

S4. TWO-QUBIT INITIALIZATION

The qubits are initialized by pulsing from the spin-blocked region, where the $|\downarrow\downarrow\rangle$-state is populated, to the (1,1) manipulation point with a linear ramp within the time t_{ramp}. By varying t_{ramp} and observing the allowed qubit transitions in a spectroscopy experiment (see Fig. S3), we identify the different initialized states. We carefully select the ramp time of $\sim 20\,\mathrm{ns}$ to ensure that only the lowest and highest energy transitions ($f_{1\uparrow}$, $f_{2\downarrow}$) are visible, indicating initialization into the $|\downarrow\uparrow\rangle$-state.

The background of the measurement shows an interference pattern. This could be explained by Landau-Zener-Stückelberg interference due to repeatedly pulsing the system across an anticrossing [3].

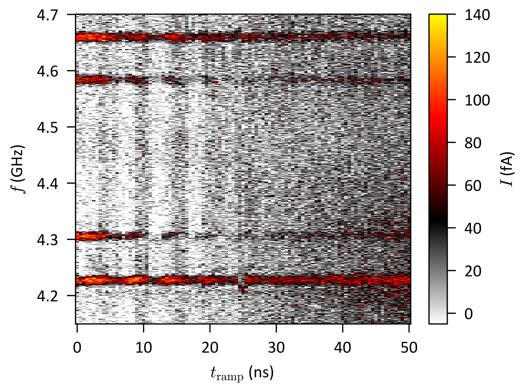

FIG. S3. **Two-qubit initialization.** MW spectroscopy measurement as a function of ramp time t_{ramp} for a trapezoid initialization and readout pulse (see Section S1). The vanishing contrast of the inner two transitions indicates an initialization into the $|\downarrow\uparrow\rangle$ state, which only allows transitions with the highest and lowest frequency. This experiment was used to calibrate $t_{\mathrm{ramp}} \sim 20\,\mathrm{ns}$ for the experiment in the main paper (see Fig. 4).

S5. DERIVATION OF EXCHANGE MATRIX FORMALISM AND FITTING FORMULA

In this section, starting from a two-site Hubbard model we derive the effective Hamiltonian of the $(1,1)$ charge sector of a double QD and show that the exchange interaction in this approximation can be written as a 3D rotation. We present the effective Hamiltonian in three different frames with the intention to assist future work on the experimental as well as theoretical side. We obtain a fitting formula for the exchange splitting as a function of magnetic field orientation, facilitating the extraction of the full exchange matrix from the MW transitions measured in the two-qubit system. Finally, we verify that in the present experiment the exchange interaction is well described by a rotation matrix, and that including an additional Ising anisotropy [4, 5], which splits triplet states at zero magnetic field, does not improve the fitting of the presented results.

We describe our double QD setup using a two-site Fermi-Hubbard model where each QD (QD1 and QD2, respectively) is described by a single orbital state $|1\rangle$ and $|2\rangle$. The Hamiltonian reads

$$H_{\mathrm{FH}} = \sum_{i,j\in\{1,2\}} \sum_{s,s'\in\{\uparrow,\downarrow\}} \tilde{H}_{ij}^{ss'} a_{is}^{\dagger} a_{js'} + U \sum_{i\in\{1,2\}} n_{i\uparrow} n_{i\downarrow}\,, \tag{3}$$

where $a_{i,s}^{\dagger}$ ($a_{i,s}$) creates (annihilates) a hole on site i with spin s, and obeys fermionic anticommutation relations. Furthermore, $n_{is} = a_{i,s}^{\dagger} a_{i,s}$ is the spin-resolved particle number operator of dot i and U is the charging energy. The single particle Hamiltonian $\tilde{H}_{ij}^{ss'} = \langle is|\,\tilde{H}\,|js'\rangle$ acting on the orbital and spin degrees of freedom reads

$$\begin{aligned}\tilde{H} =& \frac{\epsilon + U - U_0}{2}\tau_z + t\cos(\theta_{\mathrm{so}})\tau_x + t\sin(\theta_{\mathrm{so}})\tau_y \mathbf{n}_{\mathrm{so}}\cdot\boldsymbol{\sigma} \\ &+ \frac{1}{2}\mu_B \mathbf{B}\cdot\left[\frac{1+\tau_z}{2}\hat{g}_1\cdot\boldsymbol{\sigma} + \frac{1-\tau_z}{2}\hat{g}_2\cdot\boldsymbol{\sigma}\right],\end{aligned} \tag{4}$$

where τ_k are Pauli matrices acting on the orbital degrees of freedom, e.g., $\tau_z = |1\rangle\langle 1| - |2\rangle\langle 2|$, and $\boldsymbol{\sigma} = (\sigma_x, \sigma_y, \sigma_z)$ are also Pauli matrices acting on the spin degree of freedom $\{|\uparrow\rangle, |\downarrow\rangle\}$. The first term of Eq. (4) accounts for the detuning between the left and right QDs, where ϵ is measured from the singlet-singlet anticrossing. Furthermore, since the charging energy U in the experiment is measured at a different barrier height than the exchange anisotropy, we introduced U_0 as a fitting parameter that accounts for the shift of the singlet-singlet anticrossing. The tunnel-coupling between the QDs is characterized by a (spin-conserving) hopping term $\propto t\cos(\theta_{\mathrm{so}})$, while spin-orbit interaction is described by the spin-flip hopping $\propto t\sin(\theta_{\mathrm{so}})$. For spatially homogeneous SOI, the rotation angle is given by the dot-dot distance over the spin-orbit length i.e., corresponding to a spin rotation of $2\theta_{\mathrm{so}} = 2d/\lambda_{\mathrm{so}}$, and $\mathbf{n}_{\mathrm{so}}$ is the direction of the spin-orbit axis [6]. The g-tensors of the two QDs are taken into account in the most general form, where the spin quantization axis is

fixed such that the g-tensors g_1 and g_2 are symmetric if $\mathbf{B}$ is in the lab frame [7].

Spin-orbit frame. In order to eliminate the spin-flip tunnelling term and thus obtain a simpler matrix form of the Hamiltonian, we move to the *spin-orbit frame.* The corresponding unitary basis transformation is given by $\tilde{H}^{\text{so}} = U_{\text{so}}^\dagger \tilde{H} U_{\text{so}}$, where $U_{\text{so}} = \exp(-i\theta_{\text{so}}\tau_z \mathbf{n}_{\text{so}}\cdot\boldsymbol{\sigma}/2)$, which rotates the spin-quantization axes on the two sites in opposite directions. In this spin-orbit frame, the spin-conserving and spin-flip tunnelling transform as $t\cos(\theta_{\text{so}})\tau_x \to t\cos^2(\theta_{\text{so}})\tau_x - t\sin(2\theta_{\text{so}})\tau_y \mathbf{n}_{\text{so}}\cdot\boldsymbol{\sigma}/2$ and $t\sin(\theta_{\text{so}})\tau_y\mathbf{n}_{\text{so}}\cdot\boldsymbol{\sigma} \to t\sin^2(\theta_{\text{so}})\tau_x + t\sin(2\theta_{\text{so}})\tau_y\mathbf{n}_{\text{so}}\cdot\boldsymbol{\sigma}/2$, respectively, and the Hamiltonian in Eq. (4) reads

$$\tilde{H}^{\text{so}} = \frac{\epsilon + U - U_0}{2}\tau_z + t\tau_x + \frac{1}{2}\mu_B\mathbf{B}\cdot\left[\frac{1+\tau_z}{2}\hat{g}_1^{\text{so}}\cdot\boldsymbol{\sigma} + \frac{1-\tau_z}{2}\hat{g}_2^{\text{so}}\cdot\boldsymbol{\sigma}\right], \tag{5}$$

where the spin-orbit rotated g-tensors are $\hat{g}_1^{\text{so}} = \hat{g}_1\cdot\hat{R}_{\text{so}}(\theta_{\text{so}})$ and $\hat{g}_2^{\text{so}} = \hat{g}_2\cdot\hat{R}_{\text{so}}(-\theta_{\text{so}})$ with $\hat{R}_{\text{so}}(\varphi)$ denoting the right-handed rotation around the spin-orbit axis $\mathbf{n}_{\text{so}}$ by an angle φ. The transformation of the g-tensors is straightforward

$$U_{\text{so}}^\dagger\left[\frac{1+\tau_z}{2}\hat{g}_1\cdot\boldsymbol{\sigma}\right]U_{\text{so}} = \begin{pmatrix}\exp(i\theta_{\text{so}}\mathbf{n}_{\text{so}}\cdot\boldsymbol{\sigma}/2)\cdot\hat{g}_1\cdot\boldsymbol{\sigma}\exp(-i\theta_{\text{so}}\mathbf{n}_{\text{so}}\cdot\boldsymbol{\sigma}/2) & 0\\ 0 & 0\end{pmatrix} = \frac{1+\tau_z}{2}\hat{g}_1\cdot\hat{R}_{\text{so}}(\theta_{\text{so}})\cdot\boldsymbol{\sigma}, \tag{6}$$

keeping in mind the transformation rule for the vector of Pauli matrices. Note that the spin-flip tunnelling does not appear in this formulation, but the $\hat{g}_i^{\text{so}}$ matrices are not symmetric anymore.

Since the quantization axis has been rotated by $\mp\theta_{\text{so}}$ around $\mathbf{n}_{\text{so}}$ for the left and right sites, respectively, the on-site Hubbard term $Un_{i\uparrow}n_{i\downarrow}$ has the same form as in the lab frame. The Hamiltonian in Eq. (3) using the single-particle term of Eq. (5) is then projected to the lowest-energy two-particle sector using the basis states

$$|S(0,2)\rangle = a_{2\uparrow}^\dagger a_{2\downarrow}^\dagger|0\rangle, \tag{7a}$$

$$|S\rangle = \frac{1}{\sqrt{2}}(a_{1\uparrow}^\dagger a_{2\downarrow}^\dagger - a_{1\downarrow}^\dagger a_{2\uparrow}^\dagger)|0\rangle, \tag{7b}$$

$$|T_0\rangle = \frac{1}{\sqrt{2}}(a_{1\uparrow}^\dagger a_{2\downarrow}^\dagger + a_{1\downarrow}^\dagger a_{2\uparrow}^\dagger)|0\rangle, \tag{7c}$$

$$|T_{ss}\rangle = a_{1s}^\dagger a_{2s}^\dagger|0\rangle, \tag{7d}$$

where $|0\rangle$ is the vacuum state for holes and we omitted the $S(2,0)$ state since we operate close to the $S(0,2) - S$ anticrossing, i.e., $\epsilon \ll U - U_0$. The low-energy 5×5 Hamiltonian of the DQD then

reads

$$H_{5\times 5} = \begin{pmatrix} U_0-\epsilon & \sqrt{2}t & 0 & 0 & 0 \\ \sqrt{2}t & 0 & -\frac{\delta \mathrm{b}_x+i\delta \mathrm{b}_y}{\sqrt{2}} & \frac{\delta \mathrm{b}_x-i\delta \mathrm{b}_y}{\sqrt{2}} & \delta \mathrm{b}_z \\ 0 & -\frac{\delta \mathrm{b}_x-i\delta \mathrm{b}_y}{\sqrt{2}} & \bar{b}_z & 0 & \frac{\bar{b}_x-i\bar{b}_y}{\sqrt{2}} \\ 0 & \frac{\delta \mathrm{b}_x+i\delta \mathrm{b}_y}{\sqrt{2}} & 0 & -\bar{b}_z & \frac{\bar{b}_x+i\bar{b}_y}{\sqrt{2}} \\ 0 & \delta \mathrm{b}_z & \frac{\bar{b}_x+i\bar{b}_y}{\sqrt{2}} & \frac{\bar{b}_x-i\bar{b}_y}{\sqrt{2}} & 0 \end{pmatrix}, \tag{8}$$

where the order of the basis states is $[S(0,2), S, T_{\uparrow\uparrow}, T_{\downarrow\downarrow}, T_0]$ and we introduced the average- and gradient Zeeman fields as $\bar{\mathbf{b}} = (\mathbf{b}_1 + \mathbf{b}_2)/2 = \mu_B \mathbf{B} \cdot (\hat{g}_1^{\mathrm{so}} + \hat{g}_2^{\mathrm{so}})/2$ and $\delta\mathbf{b} = (\mathbf{b}_1 - \mathbf{b}_2)/2 = \mu_B \mathbf{B} \cdot (\hat{g}_1^{\mathrm{so}} - \hat{g}_2^{\mathrm{so}})/2$, where the second equality defines the Zeeman field $\mathbf{b}_i$ for the ith site. Furthermore the charging energy U of the doubly occupied singlet $S(0,2)$ is compensated by our definition of the detuning $\tilde{\epsilon} = \epsilon + U - U_0$, in Eq. (4), and U_0 remains a fitting parameter (much smaller than U).

The $[S(0,2), S]$ block of the Hamiltonian in Eq. (8) can be diagonalized exactly, leading to hybridized singlet states $[S_+, S_-]$ with $S_+ = \cos(\gamma/2)S(0,2) + \sin(\gamma/2)S$ and $S_- = -\sin(\gamma/2)S(0,2) + \cos(\gamma/2)S$ at energies $E_{S_+} = U_0 - \epsilon + J_0$ and $E_{S_-} = -J_0$, respectively, where $J_0 = \sqrt{2}t\tan(\gamma/2) = -(U_0-\epsilon)[1-\sqrt{1+8t^2/(U_0-\epsilon)^2}]/2$ and the angle $\gamma = \arctan[\sqrt{8}t/(U_0-\epsilon)]$. In the limit of large detuning $U_0 - \epsilon \gg t$ we obtain $J_0 = 2t^2/(U_0-\epsilon)$ as in Eq. (1) of the main text. After the transformation of the singlet sector one obtains

$$H_{5\times 5} = \begin{pmatrix} U_0-\epsilon+J_0 & 0 & -\frac{\delta \mathrm{b}_x+i\delta \mathrm{b}_y}{\sqrt{2}}\sin(\frac{\gamma}{2}) & \frac{\delta \mathrm{b}_x-i\delta \mathrm{b}_y}{\sqrt{2}}\sin(\frac{\gamma}{2}) & \delta \mathrm{b}_z\sin(\frac{\gamma}{2}) \\ 0 & -J_0 & -\frac{\delta \mathrm{b}_x+i\delta \mathrm{b}_y}{\sqrt{2}}\cos(\frac{\gamma}{2}) & \frac{\delta \mathrm{b}_x-i\delta \mathrm{b}_y}{\sqrt{2}}\cos(\frac{\gamma}{2}) & \delta \mathrm{b}_z\cos(\frac{\gamma}{2}) \\ -\frac{\delta \mathrm{b}_x-i\delta \mathrm{b}_y}{\sqrt{2}}\sin(\frac{\gamma}{2}) & -\frac{\delta \mathrm{b}_x-i\delta \mathrm{b}_y}{\sqrt{2}}\cos(\frac{\gamma}{2}) & \bar{b}_z & 0 & \frac{\bar{b}_x-i\bar{b}_y}{\sqrt{2}} \\ \frac{\delta \mathrm{b}_x+i\delta \mathrm{b}_y}{\sqrt{2}}\sin(\frac{\gamma}{2}) & \frac{\delta \mathrm{b}_x+i\delta \mathrm{b}_y}{\sqrt{2}}\cos(\frac{\gamma}{2}) & 0 & -\bar{b}_z & \frac{\bar{b}_x+i\bar{b}_y}{\sqrt{2}} \\ \delta \mathrm{b}_z\sin(\frac{\gamma}{2}) & \delta \mathrm{b}_z\cos(\frac{\gamma}{2}) & \frac{\bar{b}_x+i\bar{b}_y}{\sqrt{2}} & \frac{\bar{b}_x-i\bar{b}_y}{\sqrt{2}} & 0 \end{pmatrix}. \tag{9}$$

Since the couplings between S_+ and the triplet states are small, i.e. $\propto \delta b\sin(\gamma/2)$, we can restrict our Hilbert space to the lowest 4 states $\{S_-, T_{\uparrow\uparrow}, T_{\downarrow\downarrow}, T_0\}$, obtaining the effective Hamiltonian to linear order in B that accounts exactly for the tunnel coupling, as

$$H_{4\times 4} = \begin{pmatrix} -J_0 & -\frac{\delta \mathrm{b}_x+i\delta \mathrm{b}_y}{\sqrt{2}}\cos(\frac{\gamma}{2}) & \frac{\delta \mathrm{b}_x-i\delta \mathrm{b}_y}{\sqrt{2}}\cos(\frac{\gamma}{2}) & \delta \mathrm{b}_z\cos(\frac{\gamma}{2}) \\ -\frac{\delta \mathrm{b}_x-i\delta \mathrm{b}_y}{\sqrt{2}}\cos(\frac{\gamma}{2}) & \bar{b}_z & 0 & \frac{\bar{b}_x-i\bar{b}_y}{\sqrt{2}} \\ \frac{\delta \mathrm{b}_x+i\delta \mathrm{b}_y}{\sqrt{2}}\cos(\frac{\gamma}{2}) & 0 & -\bar{b}_z & \frac{\bar{b}_x+i\bar{b}_y}{\sqrt{2}} \\ \delta \mathrm{b}_z\cos(\frac{\gamma}{2}) & \frac{\bar{b}_x+i\bar{b}_y}{\sqrt{2}} & \frac{\bar{b}_x-i\bar{b}_y}{\sqrt{2}} & 0 \end{pmatrix}, \tag{10}$$

where the neglected couplings to the higher singlet only give perturbative corrections to the Hamiltonian in Eq. (10) that are $\mathcal{O}[\delta b^2/(U_0-\epsilon+J_0)]$. Therefore, in the case of sufficiently weak Zeeman

field anisotropy [$\delta b \ll (U_0 - \epsilon + J_0)$], Eq. (10) remains accurate throughout the singlet-singlet anticrossing. On the contrary, one would need to resort to perturbation theory in $t/(U_0 - \epsilon) \ll 1$, when the 5×5 Hamiltonian is written in the lab frame, causing significantly larger errors in the approximation because of the large spin-flip tunnelling terms.

From Eq. (10) it is apparent that the singlet-hybridization simply renormalizes the relative Zeeman field by a factor of $\cos(\gamma/2)$. In order to find the renormalized Zeeman fields of the left and right QDs, we write them in terms of the average and the renormalized relative fields to get $\mathbf{b}'_1 = \mathbf{b}_1 - \sin^2(\gamma/4)(\mathbf{b}_1 - \mathbf{b}_2)$ and similarly $\mathbf{b}'_2 = \mathbf{b}_2 + \sin^2(\gamma/4)(\mathbf{b}_1 - \mathbf{b}_2)$. In the cases considered in this work, $\sin^2(\gamma/4) \lesssim 0.004$, and thus we disregard the singlet-hybridization corrections and use $\mathbf{b}'_1 \approx \mathbf{b}_1$ and $\mathbf{b}'_2 \approx \mathbf{b}_2$. We note also that this approximation is rather accurate in general, because the these corrections are bounded by $\sin^2(\gamma/4) < 0.15$ since $|\gamma| < \pi/2$ by definition.

In the weak tunnelling regime $S_- \approx S(1,1)$ and the Hamiltonian of Eq. (10) is restricted to the $(1,1)$ charge sector. One can then introduce the localized spin operators $\boldsymbol{\sigma}_1^{\rm so}$, and $\boldsymbol{\sigma}_2^{\rm so}$, where the subscript 'so' refers to the spin-orbit transformation in Eq. (5). The Hamiltonian in the *spin-orbit frame* can be rewritten in terms of these operators as

$$H^{\rm so}_{(1,1)} = \frac{1}{2}\mu_B \mathbf{B} \cdot \hat{g}_1^{\rm so} \cdot \boldsymbol{\sigma}_1^{\rm so} + \frac{1}{2}\mu_B \mathbf{B} \cdot \hat{g}_2^{\rm so} \cdot \boldsymbol{\sigma}_2^{\rm so} + \frac{1}{4} J_0 \boldsymbol{\sigma}_1^{\rm so} \cdot \boldsymbol{\sigma}_2^{\rm so} \,. \tag{11}$$

Using the language of localized spin operators allows us to use simple rotations to transform the Hamiltonian *(i)* back to the lab frame, where the g-tensors are symmetric and *(ii)* to the qubit frame where the single-qubit part of the Hamiltonian is diagonal, allowing us to identify which matrix elements of the exchange matrix lead to the observed splitting.

Lab frame. The formulation of Eq. (11) facilitates to transform the effective Hamiltonian to the *lab frame* by means of real-space rotation matrices. A rotation can be applied on both left and right spin operators, independently as $\hat{R}_{\rm so}(-\theta_{\rm so}) \cdot \boldsymbol{\sigma}_1^{\rm so} = \boldsymbol{\sigma}_1$, and $\hat{R}_{\rm so}(\theta_{\rm so}) \cdot \boldsymbol{\sigma}_2^{\rm so} = \boldsymbol{\sigma}_2$. The rotations bring the g tensors back to the symmetric form, and the lab frame Hamiltonian reads

$$H^{\rm lab}_{(1,1)} = \frac{1}{2}\mu_B \mathbf{B} \cdot \hat{g}_1 \cdot \boldsymbol{\sigma}_1 + \frac{1}{2}\mu_B \mathbf{B} \cdot \hat{g}_2 \cdot \boldsymbol{\sigma}_2 + \frac{1}{4} \boldsymbol{\sigma}_1 \cdot \hat{\mathcal{J}} \cdot \boldsymbol{\sigma}_2 \,, \tag{12}$$

where $\hat{\mathcal{J}} = J_0 \hat{R}_{\rm so}(-2\theta_{\rm so})$ is the exchange matrix in the lab frame. From the nonperturbative treatment of the SOI in the two-site Hubbard model, we obtained that the anisotropy of the exchange interaction is given by a 3D rotation in accordance with Refs. [8, 9]. However, more elaborate models might lead to corrections to the exchange that cannot be written as a simple rotation matrix [5, 10]. If one would account for the effect of higher orbital states in each QD, for the

effect of a SOI cubic in momentum or the orbital effects of the magnetic field on the lowest 4×4 subspace perturbatively, the Zeeman terms would only be renormalized, but the exchange matrix could obtain additional anisotropies, e.g., Ising anisotropy. Later we consider this correction on the phenomenological level, and show that the inclusion of an additional Ising anisotropy does not significantly improve the fit and therefore we conclude that the corresponding corrections must be negligible compared to the rotational anisotropy.

Qubit frame. In order to find which matrix elements of the exchange interaction are responsible for the splitting observed in the double QD spectrum, we move to the frame where the Zeeman terms are diagonal, and consider the exchange interaction as a perturbation. Starting from the lab frame Hamiltonian of Eq. (12), using independent rotations R_1 and R_2 on Q1 and Q2, respectively the Hamiltonian can be rewritten in the *qubit frame*. In this frame the single particle terms of the Hamiltonian are diagonal, i.e.,

$$H^Q_{(1,1)} = \frac{1}{2} E_{Z,1} \sigma^Q_{z,1} + \frac{1}{2} E_{Z,1} \sigma^Q_{z,2} + \frac{1}{4} \boldsymbol{\sigma}^Q_1 \cdot \hat{\mathcal{J}}^Q \cdot \boldsymbol{\sigma}^Q_2 , \tag{13}$$

where $\mu_B \hat{R}_1 \cdot \hat{g}_1 \cdot \mathbf{B} = E_{Z,1} \mathbf{e}^Q_z$ is the Zeeman splitting on Q1 and $\mu_B \hat{R}_2 \cdot \hat{g}_2 \cdot \mathbf{B} = E_{Z,2} \mathbf{e}^Q_z$ is the Zeeman splitting of Q2, with $\mathbf{e}^Q_z$ being the qubit quantization axis. The exchange matrix in this frame incorporates also the rotations of the qubit bases, i.e., $\hat{\mathcal{J}}^Q = J_0 \hat{R}_1 \cdot \hat{R}_{\text{so}}(-2\theta_{\text{so}}) \cdot \hat{R}^T_2$. Note that the exchange matrix can still be characterized as a single rotation matrix as $\hat{\mathcal{J}}^Q = J_0 \hat{R}_{\tilde{\mathbf{n}}}(-2\tilde{\theta})$, where $\tilde{\theta}$ and $\tilde{\mathbf{n}}$ can be expressed in terms of the g-tensors, the magnetic field and the spin-orbit vectors. This frame is used here to obtain the experimentally measured exchange splitting $J_\parallel$. As it will be shown below, the splitting $J_\parallel$ is given by the diagonal matrix element of the exchange matrix $\hat{\mathcal{J}}^Q$ in the direction of the qubit quantization axis.

The exchange splitting $J_\parallel$ is defined as a difference between two transitions where one of the spins (either Q1 or Q2) is flipped while the other one is in either the $|\uparrow\rangle$ or the $|\downarrow\rangle$ state. In order to obtain an estimate for this quantity we write the Hamiltonian of Eq. (13) in the matrix notation and neglect every coupling that would contribute to the eigenvalues in $\mathcal{O}(J_0^2/E_Z)$ to obtain

$$H^Q_{(1,1)} = \begin{pmatrix} E_Z + \frac{1}{4} J^Q_{zz} & 0 & 0 & 0 \\ 0 & \frac{1}{2}\Delta E_Z - \frac{1}{4} J^Q_{zz} & \frac{1}{2} J_\perp & 0 \\ 0 & \frac{1}{2}(J_\perp)^* & -\frac{1}{2}\Delta E_Z - \frac{1}{4} J^Q_{zz} & 0 \\ 0 & 0 & 0 & -E_Z + \frac{1}{4} J^Q_{zz} \end{pmatrix} , \tag{14}$$

where the order of the basis states is $\{\uparrow\uparrow, \uparrow\downarrow, \downarrow\uparrow, \downarrow\downarrow\}$ and we defined $J_\perp = [J^Q_{xx} + J^Q_{yy} + i(J^Q_{xy} - J^Q_{yx})]/2$. In our work $J_0/E_Z \sim 0.02$, rendering the effect of the off-diagonal terms negligible. The

eigenenergies of the Hamiltonian in Eq. (14) are then simply given by

$$E_{\uparrow\uparrow} = E_Z + \frac{1}{4}J^Q_{zz}\,, \qquad E_{\downarrow\downarrow} = -E_Z + \frac{1}{4}J^Q_{zz}\,, \tag{15a}$$

$$E_{\widetilde{\uparrow\downarrow}} = \frac{1}{2}\Delta\tilde{E}_Z - \frac{1}{4}J^Q_{zz}\,, \qquad E_{\widetilde{\downarrow\uparrow}} = -\frac{1}{2}\Delta\tilde{E}_Z - \frac{1}{4}J^Q_{zz}\,, \tag{15b}$$

where $\Delta\tilde{E}_Z = \sqrt{\Delta E_Z^2 + |J_\perp|^2}$. The exchange splitting is defined as $J_\parallel = E_{\uparrow\uparrow} - E_{\widetilde{\uparrow\downarrow}} - (E_{\widetilde{\downarrow\uparrow}} - E_{\downarrow\downarrow})$ leading to $J_\parallel = J^Q_{zz}$. Writing the matrix element J^Q_{zz} in a basis-independent form we arrive at

$$J_\parallel(\mathbf{B}) = J_0\,\mathbf{e}_z \cdot \hat{R}_1 \cdot \hat{R}_{\rm so}(-2\theta_{\rm so}) \cdot \hat{R}_2^T \cdot \mathbf{e}_z = J_0\,\mathbf{n}_1 \cdot \hat{R}_{\rm so}(-2\theta_{\rm so}) \cdot \mathbf{n}_2\,, \tag{16}$$

that straightforwardly accounts for spin-orbit interaction and the anisotropy of the g-tensors. Note that $\mathbf{n}_j = \hat{g}_j \cdot \mathbf{B}/|\hat{g}_j \cdot \mathbf{B}|$ provides an explicit dependence on the magnetic field orientation for given g-tensors. The g-tensors can be related to measurable quantities (transition energies) as $E_{\uparrow\uparrow} - E_{\widetilde{\downarrow\uparrow}} + (E_{\widetilde{\uparrow\downarrow}} - E_{\downarrow\downarrow}) = 2E_Z + \Delta\tilde{E}_Z \approx 2\mu_B|\hat{g}_1 \cdot \mathbf{B}|$ and $E_{\uparrow\uparrow} - E_{\widetilde{\uparrow\downarrow}} + (E_{\widetilde{\downarrow\uparrow}} - E_{\downarrow\downarrow}) = 2E_Z - \Delta\tilde{E}_Z \approx 2\mu_B|\hat{g}_2 \cdot \mathbf{B}|$, where we use the approximation $E_{Z,1}, E_{Z,2} \gg \Delta\tilde{E}_Z - \Delta E_Z$. This approximation allows us to extract $\hat{g}$ independently from $\hat{\mathcal{J}}$, avoiding iterative processes. Hence, spectroscopy measurements for different magnetic field orientations (see Supplementary Section 2) suffice to determine the g-tensors (see Supplementary Section 3). Finally, inserting the g-tensors into eq. (16), a fitting formula is obtained that allows to straightforwardly extract the exchange matrix $\hat{\mathcal{J}}$ from the same spectroscopy measurement used to extract $\hat{g}$. In the main paper we use this formula to fit 5 independent parameters, where 3 fitting parameters are in $\hat{R}_{\rm so}(-2\theta_{\rm so})$, i.e. $\alpha_{\rm so}$, $\beta_{\rm so}$ (defining $\mathbf{n}_{\rm so}$) and $\lambda_{\rm so} = \theta_{\rm so}/d$, and 2 fitting parameters are in J_0, i.e. t and U_0.

Additional anisotropies. The relevance of the neglected higher-orbital corrections can be investigated by allowing for additional Ising anisotropy effects in the exchange interaction $\hat{\mathcal{J}}$, e.g. zero-field splitting of triplet states [4, 5]. As explained in Ref. [5], the Ising anisotropy of the exchange can be written as $\delta\hat{\mathcal{J}} = \mathcal{D}\,\mathbf{n}_{\rm so} \circ \mathbf{n}_{\rm so}$, where the anisotropy axis is the spin-orbit axis $\mathbf{n}_{\rm so}$, and $\mathcal{D}$ is the zero-field splitting. If such an effect is present, the fitting formula of Eq. (16) can be extended by the term

$$\delta J_\parallel = \mathcal{D}\,(\mathbf{n}_{\rm so} \cdot \mathbf{n}_L)(\mathbf{n}_{\rm so} \cdot \mathbf{n}_R)\,, \tag{17}$$

leading to a single fitting parameter in addition to J_0, $\mathbf{n}_{\rm so}$, and $\theta_{\rm so}$. For the present data set we obtained $\mathcal{D} = 13 \pm 2\,\text{MHz}$ for the zero-field splitting, while the other fitting parameters have

only changed within their respective error-bars. The exchange matrix and its zero-field splitting correction then read

$$\hat{\mathcal{J}} = J_0 \begin{pmatrix} -0.87 & 0.41 & -0.28 \\ -0.49 & -0.60 & 0.64 \\ 0.10 & 0.69 & 0.72 \end{pmatrix}, \qquad \delta\hat{\mathcal{J}} = J_0 \begin{pmatrix} 0.00 & 0.00 & -0.01 \\ 0.00 & 0.02 & 0.05 \\ -0.01 & 0.05 & 0.12 \end{pmatrix}. \tag{18}$$

Since the overall quality of the fit remained unchanged, we conclude that the simplified form of the exchange matrix used in the main text is indeed capturing the main source of exchange anisotropy, that is the direct Rashba SOI (linear in momentum).

S6. EXCHANGE MATRIX FOR ELECTRON QDS IN SILICON

Our analysis can be straightforwardly extended to the case of electron QDs in silicon where the SOI is induced by the gradient field of a micromagnet [11–13]. The inhomogeneous magnetic field induced by the magnet is fixed in the lab frame as opposed to the external magnetic field, the direction of which needs to be changed in order to map out the g-tensors and the exchange matrix. The low-energy Hamiltonian of such a double QD system with two-electron occupation in the $(1,1)$ charge configuration is similar to Eq. (12) but needs to be extended by the magnetic field of the micromagnets as

$$H_{(1,1)} = \frac{1}{2}\mu_B(\mathbf{B}+\mathbf{M}_1)\cdot\hat{g}_1\cdot\boldsymbol{\sigma}_1 + \frac{1}{2}\mu_B(\mathbf{B}+\mathbf{M}_2)\cdot\hat{g}_2\cdot\boldsymbol{\sigma}_2 + \frac{1}{4}\boldsymbol{\sigma}_1\cdot\hat{\mathcal{J}}\cdot\boldsymbol{\sigma}_2\,, \tag{19}$$

where $\mathbf{M}_i$ is the magnetic field induced by the micromagnet on site i, and the exchange matrix is still anisotropic due to the spin-flip tunnelling process induced by the spatially inhomogeneous magnetic field between the two QDs. In analogy with the case of SOI, the spin rotation angle can be estimated as $\tan(\theta_{\rm so}) \sim \mu_B|\mathbf{M}_1-\mathbf{M}_2|/\hbar\omega_0$, where $\hbar\omega_0$ is the orbital splitting of the QD. Because this angle is typically small, the exchange interaction is roughly isotropic, in agreement with the fact that no exchange anisotropy was reported in recent works with micromagnets [12, 13]. The strong exchange anisotropy to date is unique to hole systems with strong SOI. As it will be presented in the next section, this anisotropy can be the key to achieve fast and high-fidelity two-qubit gates for holes that keep up with the exceptionally fast single-qubit gates in these systems.

In the case of electrons, fitting the parameters of the model in Eq. (19) involves an additional step due to the field of the micromagnet. This field can be mapped out component by component, by changing the strength of the magnetic field along a given direction and determining the offset of the minimum of the Zeeman splitting with respect to $\mathbf{B} = 0$. Accounting for this fixed magnetic

field on each QD, one could proceed to fit the g-tensors and the exchange matrix as presented in Sec. S5 using $\mathbf{n}_j = \hat{g}_j \cdot (\mathbf{B} + \mathbf{M}_j)/|\hat{g}_j \cdot (\mathbf{B} + \mathbf{M}_j)|$.

S7. THEORETICAL LIMIT OF THE CNOT GATE FIDELITY

In this section we numerically calculate the fidelity of a CNOT gate, implemented via a controlled rotation (CROT) and additional correction gates. For this purpose, we extend the qubit Hamiltonian including anisotropic exchange with a driving term. Using the rotating wave approximation (RWA), we show that Rabi oscillations for Q1 can be controlled by the state of Q2. We find sequences of single- and two-qubit gates to transform a CROT into a CNOT and simulate CNOT fidelites for anisotropic and isotropic exchange interaction. We show that for anisotropic exchange and certain magnetic field orientations, the CNOT gate errors are strongly reduced in comparison to isotropic exchange and faster gate speeds are possible. Further, we show that the CNOT gate fidelity for isotropic exchange is strongly limited by $J_\perp$.

Starting from Eq. (14) we add the drive $H_{\mathrm{MW}} = \nu_R \sin(\omega_{\mathrm{MW}} t)\sigma_{x,1}$ to Q1, where $\nu_R = hf_R$ is the strength of the drive for zero frequency detuning and ω_{MW} is the frequency of the drive, and obtain

$$H^Q_{(1,1)}(t) = \begin{pmatrix} E_Z + \frac{1}{4}J_\parallel & 0 & \nu_R \sin(\omega_{\mathrm{MW}} t) & 0 \\ 0 & \frac{1}{2}\Delta E_Z - \frac{1}{4}J_\parallel & \frac{1}{2}J_\perp & \nu_R \sin(\omega_{\mathrm{MW}} t) \\ \nu_R \sin(\omega_{\mathrm{MW}} t) & \frac{1}{2}(J_\perp)^* & -\frac{1}{2}\Delta E_Z - \frac{1}{4}J_\parallel & 0 \\ 0 & \nu_R \sin(\omega_{\mathrm{MW}} t) & 0 & -E_Z + \frac{1}{4}J_\parallel \end{pmatrix}. \tag{20}$$

The gate operation that is applied to the qubits in the experiment is found by numerically calculating the time evolution of the Hamiltonian in Eq. (20)

$$\mathrm{CROT}_{\mathrm{num}} = \mathcal{T} \exp\Big[-\frac{i}{\hbar} \int_0^{t_\pi} dt H^Q_{(1,1)}(t) \Big], \tag{21}$$

where t_π is the time needed to perform a spin-flip on the target qubit and $\mathcal{T}$ indicates the time-ordered exponential. Next, we want to compare the numerically computed CROT gate operation to a perfect CNOT gate. For this purpose, we need to apply a sequence of correction gates that turn a CROT into a CNOT, which can be identified by analyzing the Hamiltonian (20) analytically.

First, we move to a rotating frame to eliminate the time-dependence in $H^Q_{(1,1)}(t)$, in which the Hamiltonian is given by

$$H_{\mathrm{rot}} = -i\hbar U^\dagger_{\mathrm{rot}} \dot{U}_{\mathrm{rot}} + U^\dagger_{\mathrm{rot}} H^Q_{(1,1)}(t)\, U_{\mathrm{rot}}, \tag{22}$$

where $U_{\rm rot}(t) = {\rm diag}[\exp(-i\omega_{\rm MW}t), 1, 1, \exp(i\omega_{\rm MW}t)]$ is the transformation between the rotating frame and the qubit frame. Using the RWA we drop the rapidly oscillating terms, e.g., $\propto \exp(-i2\omega_{\rm MW}t)$, and find

$$H_{\rm RWA} = \begin{pmatrix} E_Z + \frac{1}{4}J_\parallel - \hbar\omega_{\rm MW} & 0 & \frac{i}{2}\nu_R & 0 \\ 0 & \frac{1}{2}\Delta E_Z - \frac{1}{4}J_\parallel & \frac{1}{2}J_\perp & \frac{i}{2}\nu_R \\ -\frac{i}{2}\nu_R & \frac{1}{2}(J_\perp)^* & -\frac{1}{2}\Delta E_Z - \frac{1}{4}J_\parallel & 0 \\ 0 & -\frac{i}{2}\nu_R & 0 & -E_Z + \frac{1}{4}J_\parallel + \hbar\omega_{\rm MW} \end{pmatrix}. \tag{23}$$

Then, we transform to the eigenbasis of the Hamiltonian (14). This transformation accounts for the mixing of $|\uparrow\downarrow\rangle$ and $|\downarrow\uparrow\rangle$ basis states by $J_\perp$ and is defined as $\tilde{H}_{\rm RWA} = U^\dagger_{\phi,\xi} H_{\rm RWA} U_{\phi,\xi}$, where the transformation matrix is given by

$$U_{\phi,\xi} = \begin{pmatrix} 1 & 0 & 0 & 0 \\ 0 & \cos\frac{\phi}{2} & -e^{-i\xi}\sin\frac{\phi}{2} & 0 \\ 0 & e^{i\xi}\sin\frac{\phi}{2} & \cos\frac{\phi}{2} & 0 \\ 0 & 0 & 0 & 1 \end{pmatrix}, \tag{24}$$

with $\exp(i\xi) = J_\perp/|J_\perp|$ and the mixing angle $\phi = \arctan(|J_\perp|/\Delta E_Z)$. Note that this transformation commutes with $U_{\rm rot}(t)$. We obtain

$$\tilde{H}_{\rm RWA} = \begin{pmatrix} -\frac{1}{2}\Delta\tilde{E}_Z - \frac{1}{4}J_\parallel & \frac{i}{2}e^{i\xi}\nu_R\sin\frac{\phi}{2} & \frac{i}{2}\nu_R\cos\frac{\phi}{2} & 0 \\ -\frac{i}{2}e^{i\xi}\nu_R\sin\frac{\phi}{2} & \frac{1}{2}\Delta\tilde{E}_Z - \frac{1}{4}J_\parallel & 0 & \frac{i}{2}\nu_R\cos\frac{\phi}{2} \\ -\frac{i}{2}\nu_R\cos\frac{\phi}{2} & 0 & -\frac{1}{2}\Delta\tilde{E}_Z - \frac{1}{4}J_\parallel & -\frac{i}{2}e^{-i\xi}\nu_R\sin\frac{\phi}{2} \\ 0 & -\frac{i}{2}\nu_R\cos\frac{\phi}{2} & \frac{i}{2}e^{-i\xi}\nu_R\sin\frac{\phi}{2} & \frac{1}{2}\Delta\tilde{E}_Z + \frac{3}{4}J_\parallel \end{pmatrix}, \tag{25}$$

where we substituted the resonance condition for the transition that we want to drive, i.e. $|\downarrow\uparrow\rangle \to |\uparrow\uparrow\rangle$, as $\hbar\omega_{\rm MW} = E_Z + \frac{1}{2}\Delta\tilde{E}_Z + \frac{1}{2}J_\parallel$. We note that, depending on the sign of $J_\parallel$, we obtain a CROT or a not-controlled rotation (NCROT). In the RWA Hamiltonian we call the off-diagonal terms that connect degenerate states *resonant* transitions, i.e. $|\downarrow\uparrow\rangle \to |\uparrow\uparrow\rangle$, whereas terms connecting two states that are not degenerate are the *off-resonant* transitions. Off-resonant transitions are highly suppressed by the energy mismatch, hence we neglect all off-resonant terms. Note that off-resonant terms that include $\sin(\phi/2)$ vanish completely for $\phi = 0$, i.e. $J_\perp = 0$, reducing the error introduced by the approximation for this specific case.

Next, we calculate the complete time evolution of the qubit states. Within the rotating frame and the RWA, the time evolution of a state in the qubit frame is given by $|\psi(t)\rangle = U_{\rm rot}(t)U_{\rm RWA}(t)|\psi(0)\rangle$,

where $U_{\text{RWA}}(t)$ is the free time evolution according to the RWA Hamiltonian (23). Because $U_{\phi,\xi}$ commutes with the rotating frame transformation U_{rot}, one may write $U_{\text{rot}}(t)U_{\text{RWA}}(t) = U_{\phi,\xi}U_{\text{rot}}(t)\exp(-i/\hbar\tilde{H}_{\text{RWA}}t)U^{\dagger}_{\phi,\xi} = U_{\phi,\xi}U_{\text{rot}}(t)\tilde{U}_{\text{RWA}}(t)U^{\dagger}_{\phi,\xi}$. The full time evolution under the Hamiltonian in the mixed basis is then

$$\begin{aligned}\widetilde{\text{CROT}} =& U_{\text{rot}}(t_\pi)\tilde{U}_{\text{RWA}}(t_\pi) = \\ & \begin{pmatrix} 0 & 0 & e^{i\pi\kappa\left(-\frac{E_Z}{J_\parallel}-\frac{1}{4}\right)} & 0 \\ 0 & e^{i\pi\kappa\left(-\frac{\Delta\tilde{E}_Z}{2J_\parallel}+\frac{1}{4}\right)} & 0 & 0 \\ -e^{i\pi\kappa\left(\frac{\Delta\tilde{E}_Z}{2J_\parallel}+\frac{1}{4}\right)} & 0 & 0 & 0 \\ 0 & 0 & 0 & e^{i\pi\kappa\left(\frac{E_Z}{J_\parallel}-\frac{1}{4}\right)} \end{pmatrix},\end{aligned} \tag{26}$$

where the operation time for a π-rotation is $t_\pi = h/(2\nu_R\cos(\phi/2))$ and we imposed $\nu_R\cos(\phi/2) = J_\parallel/\kappa$ with $\kappa = \sqrt{16k^2-1}$ and k is an integer as in Ref. [14]. These conditions restrict the maximal driving strength to $\nu_R = J_\parallel/\sqrt{15}$, but ensure that no net spin rotation of Q1 occurs for the $|\downarrow\rangle$-state of the control qubit Q2. This is standard practise to reduce fidelity loss due to off-resonant driving effects [12].

The controlled rotation in the mixed basis in Eq. (26) is now compared to an ideal CNOT, which is controlled by Q2 and targeted on Q1, in the basis $\{\uparrow\uparrow, \uparrow\downarrow, \downarrow\uparrow, \downarrow\downarrow\}$

$$\text{CNOT} = \begin{pmatrix} 0 & 0 & 1 & 0 \\ 0 & 1 & 0 & 0 \\ 1 & 0 & 0 & 0 \\ 0 & 0 & 0 & 1 \end{pmatrix}. \tag{27}$$

This allows us to find a sequence of elementary single-qubit gates such that

$$\widetilde{\text{CROT}} = e^{i\pi(\Delta E_Z/2\nu_R + J_\parallel/4\nu_R - E_{Z,1}/\nu_R + 3/2)}\, Z_2^{E_{Z,2}/\nu_R+1/2}\, Z_1^{E_{Z,1}/\nu_R-1/2}\, \text{CNOT}\, Z_1^{-J_\parallel/2\nu_R+1/2}. \tag{28}$$

Here, Z_i is the Z-gate with the convention $Z_i^a = \text{diag}(1, e^{i\pi a})$ acting on the ith qubit. We consider the gate Z_i^a as directly accessible for spin qubits, since arbitrary Z-rotations can be implemented e.g. by an arbitrary detuning pulse [1, 15] or by virtual phase gates [12]. Note that the decomposition into correction gates is not unique.

If $U_{\phi,\xi} = 1$ at $\phi = 0$, hence the mixed basis is equal to the qubit basis, the CNOT gate can be constructed from the CROT gate using Eq. (28). However, having a finite mixing angle ϕ,

i.e. $J_\perp \neq 0$, we also have to account for the additional transformation $\text{CROT} = U_{\phi,\xi}\,\widetilde{\text{CROT}}\,U^\dagger_{\phi,\xi}$. Hence, we have to decompose $U_{\phi,\xi}$ into elementary gates

$$U_{\phi,\xi} = Z_1^{-\xi/\pi-1/2}\, X_1\,(\text{CZ})^{\phi/2\pi}\, X_1\, X_2\,(\text{CZ})^{\phi/2\pi}\, X_2\,(\text{SWAP})^{-\phi/\pi}\, Z_1^{\xi/\pi+1/2}, \tag{29}$$

where the controlled-Z gate (CZ) is controlled by Q1 and targeted on Q2 and X_i is the X-gate acting on the ith qubit. Note that this decomposition contains in addition to elementary single-qubit gates also multiple two-qubit gates. Introducing additional two-qubit gates creates new sources for errors, that can lower the overall-fidelity of the CNOT gate. Additionally, this creates a large overhead of correction gates, making it desirable to work in the regime of $\Delta E_Z \gg J_\perp$, where $U_{\phi,\xi} \approx 1$. Further, since SWAP and CZ gates typically require opposite regimes of $\Delta E \ll J_\perp$ and $\Delta E \gg J_\perp$, these correction gates are not practical in any experimental realization and will only be considered here to investigate the sources of errors.

Finally, we define CNOT_num as the numerically simulated CROT gate from Eq. (21) after applying single-qubit correction gates as described in Eq. (28). The fidelity of this two-qubit gate is then calculated by comparing it to the ideal CNOT gate:

$$\mathcal{F} = \frac{1}{4}\left|\text{Tr}\left[\text{CNOT}_\text{num}\text{CNOT}^\dagger\right]\right|, \tag{30}$$

Analogously, we define $\text{CNOT}^{\phi,\xi}_\text{num}$ as the numerically simulated CROT gate from Eq. (21) after applying both the single-qubit correction gates from Eq. (28) as well as the single- and two-qubit correction gates from eq. (29) and calculate the fidelity analogously.

We present numerical simulations of the fidelity in Fig. S4 for four different cases, which differ by the exchange interaction (isotropic vs anisotropic exchange) and the correction gates that are applied (only single-qubit corrections or both correction sequences). The simulation is performed by calculating the time evolution operator U with the full time-dependence of Eq. (21). Due to discretization of time, a small numerical error caused by the numerical precision is added in every time step ($dt = 10^{-7}$ ns). This contribution is not unitary and leads to $|\text{Tr}[U^\dagger U]/4| \lesssim 1$. Therefore, we associate $1 - |\text{Tr}[U^\dagger U]/4|$ with the precision of our calculations and plot it as error bar in Fig. S4.

We present the CNOT fidelity as a function of $J_\parallel/\Delta E_Z$. Since $J_\parallel = \sqrt{15}\nu_R$ was fixed for maximal driving strength without inducing unwanted off-resonant driving, this can be seen as evaluating the fidelity as a function of gate speed. We note that for a small gate speed, requiring more time steps, the error bars are increased. Overall, we see a drop of fidelity with gate speed, which is much more pronounced for isotropic than anisotropic exchange. For the case of isotropic exchange, the fidelity drops rapidly for large $J_\parallel/\Delta E_Z$, even when applying all correction gates ($\text{CNOT}^{\phi,\xi}_\text{num}$). This loss of

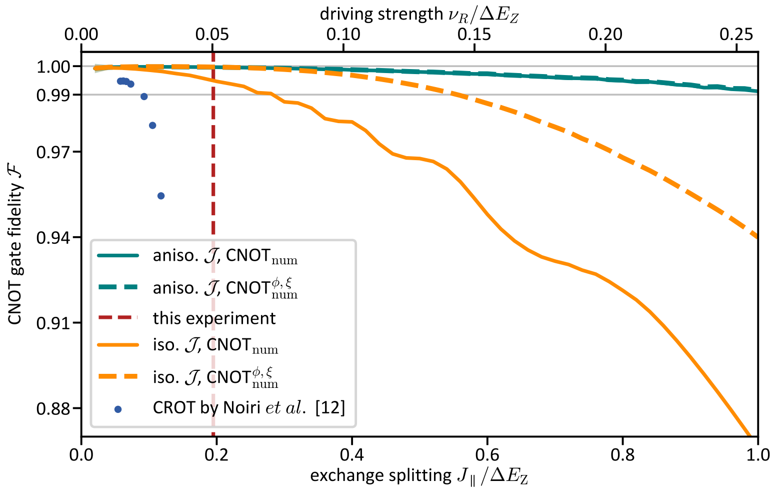

FIG. S4. CNOT gate fidelities as a function of $J_\parallel$ and driving strength ($\nu_R = J_\parallel/\sqrt{15}$) using (i) anisotropic exchange interaction (green) for the configuration used in Fig. 4; (ii) and isotropic exchange interaction (orange) e.g. for electrons in silicon. CNOT gates obtained using single-qubit correction gates only ($\mathrm{CNOT_{num}}$) are shown as solid lines, while CNOT gates also corrected for basis mixing errors ($\mathrm{CNOT_{num}^{\phi,\xi}}$) are shown as dashed lines. Red line and blue points indicate the working point of the present experiment and fidelities measured in Ref. [12], respectively. The horizontal gray line marks a fidelity of $\mathcal{F} = 99\%$. The shaded regions indicate the precision of the numerics.

fidelity can be understood as the effect of the off-resonant terms that were neglected in eq. (25), which become relevant at large driving strength. When looking at isotropic exchange and only single-qubit correction gates ($\mathrm{CNOT_{num}}$), we see a further reduction of fidelity, which originates from the strong mixing of qubit basis states due to large $J_\perp = J_\parallel$. This is the dominant effect for the loss of fidelity of the CNOT gate at small driving speeds $J_\parallel/\Delta E_Z < 0.5$. Note that the wiggle features in the fidelity probably originate from an interplay of the single-qubit correction gates and the unwanted effects of $U_{\phi,\xi}$ that are not corrected here. Comparing the theoretical fidelity to current experimental realizations of CROT gates with isotropic exchange, e.g. Noiri *et al.* [12], we find that the fidelity seem to be limited mainly by the experimental implementation. Further, these experiments are performed at very small driving strength, where the maximum theoretical CNOT fidelity is not significantly limiting the fidelity of the implemented gate.

In the anisotropic case, the fidelity depends on the magnetic field orientation, since it determines $J_\perp$. Here, we look at the case of the magnetic field orientation and $\mathcal{J}$ of the CROT experiment in Fig. 4 of the main paper. In Fig. S5 we show $J_\parallel$ and $J_\perp$ as a function of magnetic field orientation and indicate the orientation that was used with a red star, showing a large $|J_\parallel| = 0.902 J_0$ and small $|J_\perp| = 0.049 J_0$. We note that there is a large range of orientations, where such a

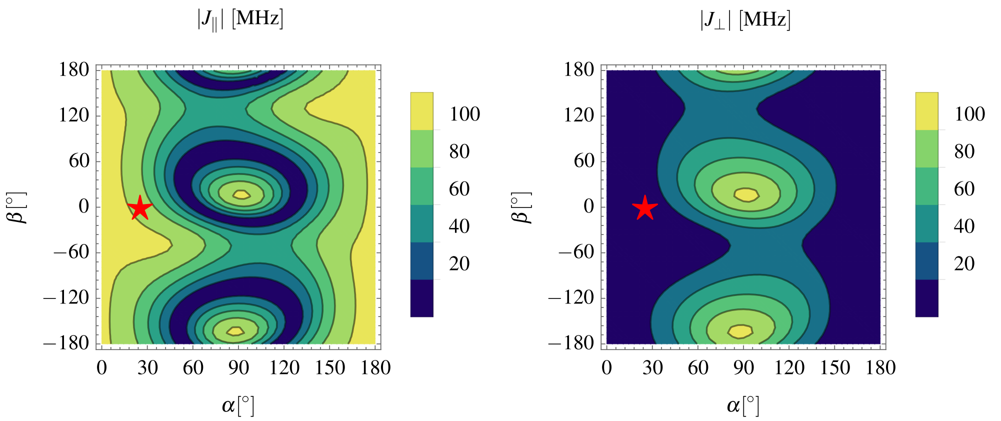

FIG. S5. Exchange interaction $|J_\parallel|$ and $|J_\perp|$ as a function of magnetic field orientation (α, β), using the experimental g-tensors, spin rotation angle θ_{so} and SOI orientation $\mathbf{n}_{so}$. Red stars denote the magnetic field direction used for the implementation of the CROT gate in the main paper and for calculating the gate fidelity in Fig. S4.

combination of $|J_\parallel|/|J_\perp| \gg 1$ can be found. In this case, the fidelity stays above 99% up to strong driving of $J_\parallel/\Delta E_Z \sim 1$. There is almost no difference between $\mathrm{CNOT}_{\mathrm{num}}$ and $\mathrm{CNOT}^{\phi,\xi}_{\mathrm{num}}$, indicating that the basis mixing by $U_{\phi,\xi}$ is not limiting the fidelity. This is expected, since for the chosen magnetic field orientation $|J_\perp| \ll \Delta E_Z$ and thus $U^{\phi,\xi} \sim 1$. The main reduction in fidelity originates from neglecting the off-resonant terms and the rapidly oscillating terms in the RWA. Hence, two-qubit correction gates, which are relevant in the isotropic case already at small driving strength, are not needed here. For much stronger driving $J_\parallel/\Delta E_Z \gtrsim 0.5$ the benefits for fidelity of anisotropic exchange become even stronger: In this regime, even when using the impractical two-qubit correction gates, the fidelity for isotropic exchange is limited to much smaller values than for anisotropic exchange.

The red dashed line in Fig. S4 indicates the value of $J_\parallel/\Delta E_Z \sim 0.2$ that was used in this experiment, showing that the fidelity of our CROT implementation is not significantly limited by the maximum theoretical fidelity. However, for isotropic exchange this diving strength would already induce a significant reduction of fidelity, unless the very hard to realize and computationally demanding two-qubit correction gates are implemented. Hence, this experiment already benefits from the anisotropic exchange interaction.

S8. ADDITIONAL INFORMATION TO FIG. 2

In this section, we present additional information to Fig. 2 of the main paper, where Eq. 1 is fitted to measurement data. The lever arms for the plunger gates are $\alpha_{\mathrm{P1}}^{\mathrm{Q1}} = 0.12$ and $\alpha_{\mathrm{P2}}^{\mathrm{Q2}} = 0.06$, while the lever arms for the barrier gates are $\alpha_{\mathrm{B}}^{\mathrm{Q1}} = 0.21$ and $\alpha_{\mathrm{B}}^{\mathrm{Q2}} = 0.21$. A charging energy of $U = 13.2\,\mathrm{meV}$ was extracted from a charge stability measurement. In our fitting routine, for each barrier gate voltage an energy offset U_0 was fitted, capturing the electric tunability of the charging energy. U_0 as function of V_B is presented in Fig. S6.

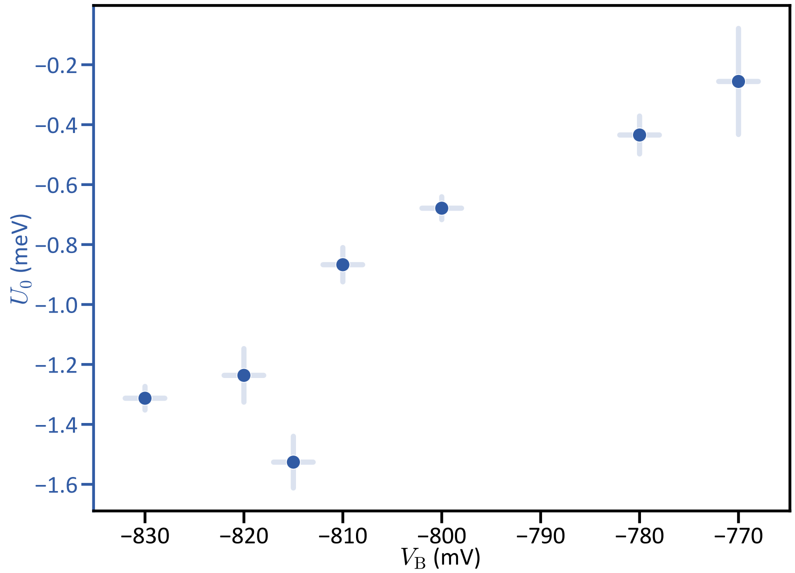

FIG. S6. Fitting parameter U_0 as function of V_B. The V_B error bars represent the estimated errors due to a device tuning uncertainty, and the U_0 error bars the standard errors for the best-fit values.

[1] L. C. Camenzind, S. Geyer, A. Fuhrer, R. J. Warburton, D. M. Zumbühl, and A. V. Kuhlmann, A hole spin qubit in a fin field-effect transistor above 4 kelvin, Nature Electronics **5**, 178 (2022).

[2] A. Crippa, R. Maurand, L. Bourdet, D. Kotekar-Patil, A. Amisse, X. Jehl, M. Sanquer, R. Laviéville, H. Bohuslavskyi, L. Hutin, S. Barraud, M. Vinet, Y.-M. Niquet, and S. D. Franceschi, Electrical Spin Driving by g-Matrix Modulation in Spin-Orbit Qubits, Physical Review Letters **120**, 137702 (2018).

[3] S. Shevchenko, S. Ashhab, and F. Nori, Landau–Zener–Stückelberg interferometry, Physics Reports **492**, 1 (2010).

[4] G. Katsaros, J. Kukučka, L. Vukušić, H. Watzinger, F. Gao, T. Wang, J.-J. Zhang, and K. Held, Zero Field Splitting of Heavy-Hole States in Quantum Dots, Nano Letters **20**, 5201 (2020).

[5] B. Hetényi, S. Bosco, and D. Loss, Anomalous Zero-Field Splitting for Hole Spin Qubits in Si and Ge Quantum Dots, Physical Review Letters **129**, 116805 (2022).

[6] F. N. M. Froning, M. J. Rančić, B. Hetényi, S. Bosco, M. K. Rehmann, A. Li, E. P. A. M. Bakkers, F. A. Zwanenburg, D. Loss, D. M. Zumbühl, and F. R. Braakman, Strong spin-orbit interaction and g-factor renormalization of hole spins in Ge/Si nanowire quantum dots, Physical Review Research **3**, 013081 (2021).

[7] Note that *(i)* g_i must be a real matrix to ensure hermiticity, *(ii)* under a general unitary transformation, the Pauli matrices transform as $U^\dagger(\sigma_x, \sigma_y, \sigma_z)U = R_{ij}(\sigma_x, \sigma_y, \sigma_z)_j$, where R is a 3D rotation. Exploiting the ambiguity of the basis choice in the Hilbert space, we define the quantization axis in the lab frame such that the g-tensor is a symmetric matrix for a given $\mathbf{B}$.

[8] K. V. Kavokin, Anisotropic exchange interaction of localized conduction-band electrons in semiconductors, Physical Review B **64**, 075305 (2001).

[9] K. V. Kavokin, Symmetry of anisotropic exchange interactions in semiconductor nanostructures, Physical Review B **69**, 075302 (2004).

[10] B. Hetényi, C. Kloeffel, and D. Loss, Exchange interaction of hole-spin qubits in double quantum dots in highly anisotropic semiconductors, Physical Review Research **2**, 033036 (2020).

[11] M. Pioro-Ladrière, T. Obata, Y. Tokura, Y.-S. Shin, T. Kubo, K. Yoshida, T. Taniyama, and S. Tarucha, Electrically driven single-electron spin resonance in a slanting Zeeman field, Nature Physics **4**, 776 (2008).

[12] A. Noiri, K. Takeda, T. Nakajima, T. Kobayashi, A. Sammak, G. Scappucci, and S. Tarucha, Fast universal quantum gate above the fault-tolerance threshold in silicon, Nature **601**, 338 (2022).

[13] X. Xue, M. Russ, N. Samkharadze, B. Undseth, A. Sammak, G. Scappucci, and L. M. K. Vandersypen, Quantum logic with spin qubits crossing the surface code threshold, Nature **601**, 343 (2022).

[14] M. Russ, D. M. Zajac, A. J. Sigillito, F. Borjans, J. M. Taylor, J. R. Petta, and G. Burkard, High-fidelity quantum gates in Si/SiGe double quantum dots, Physical Review B **97**, 085421 (2018).

[15] J. Yoneda, K. Takeda, T. Otsuka, T. Nakajima, M. R. Delbecq, G. Allison, T. Honda, T. Kodera, S. Oda, Y. Hoshi, N. Usami, K. M. Itoh, and S. Tarucha, A quantum-dot spin qubit with coherence limited by charge noise and fidelity higher than 99.9%, Nature Nanotechnology **13**, 102 (2017).
